# Intratumor microbiome derived glycolysis-lactate signatures depicts immune heterogeneity in lung adenocarcinoma by integration of microbiomic, transcriptomic, proteomic and single-cell data

**DOI:** 10.3389/fmicb.2023.1202454

**Published:** 2023-08-17

**Authors:** Xiaheng Deng, Xiru Chen, Yu Luo, Jun Que, Liang Chen

**Affiliations:** Department of Thoracic Surgery, The First Affiliated Hospital of Nanjing Medical University, Nanjing, China

**Keywords:** lung adenocarcinoma, cancer microbiome, glycolysis, prognosis, immune microenvironment

## Abstract

**Introduction:**

Microbiome plays roles in lung adenocarcinoma (LUAD) development and anti-tumor treatment efficacy. Aberrant glycolysis in tumor might promote lactate production that alter tumor microenvironment, affecting microbiome, cancer cells and immune cells. We aimed to construct intratumor microbiome score to predict prognosis of LUAD patients and thoroughly investigate glycolysis and lactate signature’s association with LUAD immune cell infiltration.

**Methods:**

The Cancer Genome Atlas-LUAD (TCGA-LUAD) microbiome data was downloaded from cBioPortal and analyzed to examine its association with overall survival to create a prognostic scoring model. Gene Set Enrichment Analysis (GSEA) was used to find each group’s major mechanisms involved. Our study then investigated the glycolysis and lactate pattern in LUAD patients based on 19 genes, which were correlated with the tumor microenvironment (TME) phenotypes and immunotherapy outcomes. We developed a glycolysis-lactate risk score and signature to accurately predict TME phenotypes, prognosis, and response to immunotherapy.

**Results:**

Using the univariate Cox regression analysis, the abundance of 38 genera were identified with prognostic values and a lung-resident microbial score (LMS) was then developed from the TCGA-LUAD-microbiome dataset. Glycolysis hallmark pathway was significantly enriched in high-LMS group and three distinct glycolysis-lactate patterns were generated. Patients in Cluster1 exhibited unfavorable outcomes and might be insensitive to immunotherapy. Glycolysis-lactate score was constructed for predicting prognosis with high accuracy and validated in external cohorts. Gene signature was developed and this signature was elevated in epithelial cells especially in tumor mass on single-cell level. Finally, we found that the glycolysis-lactate signature levels were consistent with the malignancy of histological subtypes.

**Discussion:**

Our study demonstrated that an 18-microbe prognostic score and a 19-gene glycolysis-lactate signature for predicting prognosis of LUAD patients. Our LMS, glycolysis-lactate score and glycolysis-lactate signature have potential roles in precision therapy of LUAD patients.

## Background

Despite the improvements in the treatment of lung adenocarcinoma (LUAD) in the recent decades, the 5-year survival rate remains below satisfactory levels (<20%) (Reck et al., 2022). Presently, both cellular and humoral biomarkers are extensively employed to predict the emergence and prognosis of LUAD. Advancements in next-generation sequencing (NGS) technology have led researchers to primarily investigate genomic and proteomic alterations in order to identify early detection markers, including CEA (Grunnet and Sorensen, 2012), SHOX2/PTGER4 methylation (Weiss et al., 2017; Schotten et al., 2021), microRNAs (miRNAs) and circulating tumor DNAs (ctDNAs) (Du et al., 2018; Seijo et al., 2019; Xia et al., 2022). However, emerging studies reveal that NGS profiles contain an enormous amount of microbial-derived sequencing reads that might offer new insights into the tumorigenesis markers (Poore et al., 2020).

The shifts in microbiota community structure might contribute to carcinogenesis and affect anti-cancer therapies through multiple biological pathways, such as immune, metabolism and signal transduction (Garrett, 2015). Studies have proved that malignancies such as colorectal, gastric, hepatocellular and pancreatic cancers are closely related to the microbiota (Vogtmann and Goedert, 2016; Mima et al., 2017). The well-known presence of *Helicobacter pylori* in the upper gastrointestinal tract was closely associated with stomach ulcers and elevated risk of gastric cancer (Burkitt et al., 2017). *Citrobacter rodentium* was found to facilitate colon cancer progression in mouse models (Atarashi et al., 2015). The lungs provide the largest interface for gas exchange, which are inevitably exposed to diversified environmental microorganisms. *Proteobacteria*, *Bacteroidetes*, *Firmicutes*, and *Actinobacteria* are found to be the major phyla of the healthy lung microbiome (Charlson et al., 2011). In patients with acute or chronic pulmonary diseases, such as chronic obstructive pulmonary disease and asthma, the micro-anatomic alterations in bacterial colonization in lungs could even be observed before CT scan showed abnormalities (Erb-Downward et al., 2011). There is growing evidence supporting a central role for microbiome in the development of lung cancer (Pilaniya et al., 2016; Yu et al., 2016). Greathouse et al. identified a correlation between *Acidovorax*, a bacterium associated with smoking, and the progression of lung squamous cell carcinoma harboring TP53 mutations (Greathouse et al., 2018). In addition, the combination of *Capnocytophaga* and *Veillonella* exhibited promising results for predicting the malignancy of non-small cell lung cancer and have the potential to serve as novel biomarkers (Yan et al., 2015). Recently emerged studies also showed that disturbances of microbiome composition such as antibiotics use might impair the efficacy of antitumor immunotherapy (Botticelli et al., 2020). For example, *Bifidobacterium* was discovered by Sivan et al. (2015) to increase anti-PD-L1 activity in germ free mice whereas anti-CTLA-4 response was impacted in antibiotic mice. Thus, the recognition of microbiome in LUAD cancers could predict the prognostic status of individual patient.

To this end, our study embarked on a comprehensive investigation into the distribution of intratumor microbes and their association with glycolysis-lactate clusters in LUAD patients. We aimed to establish a microbial abundance-derived prognostic score, the lung resident microbe score (LMS), and to evaluate its relationship with the prognosis and immune infiltration of LUAD. Subsequently, we applied Gene Set Enrichment Analysis (GSEA) HallMark pathways enrichment to the high LMS group, which led to the discovery of the glycolysis hall pathway. This finding prompted us to develop glycolysis-lactate based patterns and evaluate their function in LUAD prognosis. As a result, we constructed a glycolysis-lactate score, validated as a robust tool for further exploring the role of these patterns in the immune microenvironment and drug sensitivity. Through this comprehensive approach, we aimed to deepen the understanding of the connection between the tissue microbiome and metabolic changes in cancer development.

## Materials and methods

### Sample datasets and clinical profiles collected for analysis

The RNA sequence data, clinical data, microbiome data of TCGA-LUAD were obtained from the cBioPortal.^1^ Fragments Per Kilobase Million (FPKM) values were converted to Transcripts Per Million (TPM) values using TPM = (FPKM/FPKM_sum_) × 10^6^. The TCGA-LUAD-microbial profile, which was classified at the genus level rather than the species level, was generated by Dohlman et al. as a part of The Cancer Microbiome Atlas (TCMA) (Dohlman et al., 2021). After removing duplicates and patients without follow-up information, RNA-sequence data from 522 cases and microbiome data from 491 cases were enrolled in this study. We included four Gene Expression Omnibus (GEO) RNA-sequence datasets^2^ (GSE40791 (Zhang et al., 2012), GSE46539 (Chen et al., 2015), GSE72094 (Schabath et al., 2016) and GSE115002 (Cui et al., 2019)) for establishing the prognostic model, with GSE72094 containing complete survival information (Schabath et al., 2016). The “Combat” function in the “sva” R package was utilized to remove batch effects among the TCGA datasets and GEO microarrays. Additionally, we used GSE31210, which contains 246 cases with overall survival (OS) and progression free survival (PFS) data, as an external validation cohort. Furthermore, we downloaded the proteome profile PMID35383171 from the supplementary data of Mirhadi et al.’s (2022) study. In addition, GSE131907, which is a single-cell RNA sequencing dataset for LUAD, was utilized to validate the prognostic model at the single-cell level. Moreover, GSE58772, the only dataset featuring histological subtype classifications of LUAD, enabled us to investigate the potential of our prognostic model in distinguishing these subtypes (Table 1).

**Table 1:** Summary of publicly available data utilized in the study.

Data set	Data type	Glycolysis-lactate score construction cohort
TCGA-LUAD-microbiome	Intratumor microbiome	
PMID35383171	Proteome	
GSE131907	scRNA-seq	
GSE40791	RNA-seq	
GSE46539	RNA-seq	
GSE115002	RNA-seq	
GSE58772	RNA-seq	
GSE72094	RNA-seq	Training cohort
TCGA-LUAD-RNAseq	RNA-seq	Internal validation cohort
GSE31210	RNA-seq	External validation cohort

### Development of lung-resident microbial score (LMS) and clinical nomogram

The lung-resident microbial score was generated through specific procedures. The TCGA-LUAD-microbiome dataset was split into a training cohort and test cohort in a 1:1 ratio using the “createDataPartition” method available in the “caret” R package. Microbial taxa significantly associated with OS (*p* < 0.05) were identified using univariate Cox regression analysis in the training cohort. These microbes were categorized as protectors (hazard ratio (HR) <1) or risk-factors (HR >1). The least absolute shrinkage and selection operator (LASSO) regression analysis, performed using the “glmnet” R package, was used to further filter candidate microbes. To develop the LMS for the 18 selected microbes (i) with the best predictive performance, we used the following formula: LMS= ∑Coefficienti×Abundancei . The coefficients for each microbe were derived *via* multivariate Cox regression analysis. To partition the LMS, we arranged the patients in the validation set as either High-LMS or Low-LMS based on the median LMS value. The prognostic performance of the LMS was assessed using the Kaplan–Meier (K-M) method and log-rank test available in the “survminer” R package. The “timeROC” function from the “tROC” R package and principal component analysis (PCA) were used to evaluate the predictive accuracy and stability of LMS. The test cohort was used to further verify the feasibility of LMS. The “pheatmap” R package was used to plot survival curves and display the risk score distribution.

To determine if the LMS was an independent prognostic parameter, multivariate Cox regression analysis was performed, with clinicopathological factors, identified as significant in univariate analysis, including age, sex, and stage. Using the “rms” R package, a nomogram was constructed that incorporated clinical characteristics and LMS, to predict patient survival probability at 1, 3, and 5 years. To evaluate the accuracy of the nomogram-predicted survival rates, calibration plots were generated to compare the predicted versus actual survival rates.

### Primary exploration of immunity and GSEA in LMS groups

To elucidate the discrepancy between the High-LMS and Low-LMS patients, immunocyte infiltration was analyzed based on the CIBERSORT algorithms^3^ from TCGA-LUAD-transcriptome database and the subtle differences were visualized by radar chart using the “ggpubr” R package. Moreover, in view of the major mechanisms involved the High-and Low-LMS groups remained unclear, hallmark pathways were recognized using “h.all.v2022.1.Hs.symbols.gmt” gene set from MsigDB website.^4^ The “clusterProfiler” and the “enrichplot” R packages were used for the enrichment analysis with the transcriptomic data of individual samples.

### Glycolysis and lactate metabolic signatures and unsupervised clustering to identify LUAD subtypes

To quantify glycolysis and lactate metabolic enrichment scores in the High-and Low-LMS groups, we used 13 gene sets related to glycolysis and lactate metabolism recruited from the GSEA database. We employed single-sample GSEA (ssGSEA) using the “GSVA” R package to calculate these scores. Consensus clustering was performed on the merged TCGA-GEO dataset by utilizing ssGSEA scores for each sample. The R package “ConsensusClusterPlus” was implemented for repeating the process 1,000 times. The parameters for the analysis were set to maxK = 5, pItem = 0.8, reps = 1,000, clusterAlg = “pam,” and distance = “euclidean.”

### Antitumor immunity depiction of LUAD patients

In this study, we analyzed the tumor immune microenvironment (TIME) of LUAD, which comprises various components, including the infiltration of immune cells, immune and stromal scores, analysis of immune-related signatures, and anti-cancer immunity. To quantify the activity of anti-cancer immunity across the seven-step cancer-immunity cycle, we used the tracking tumor immunophenotype (TIP) database^5^ (Xu et al., 2018). We determined the abundance of 28 immunocytes infiltrating the TCGA-GEO merged dataset using the ssGSEA algorithm and previously established marker gene sets, as reported by Charoentong et al. (2017). Stromal and immune scores in TIME were estimated using the ESTIMATE algorithm (Yoshihara et al., 2013). We also calculated enrichment scores for four immune-related signatures, previously reported by Braun et al., using the ssGSEA method. Furthermore, we evaluated the potential effectiveness of immune checkpoint blockade (ICB) therapy in each sample using 21 signatures from Mariathasan et al.’s study (Mariathasan et al., 2018; Braun et al., 2020). In addition, we utilized the Tumor Immune Dysfunction and Exclusion (TIDE) algorithm^6^ to predict the immunotherapeutic response of each patient with LUAD (Jiang et al., 2018).

### Drug sensitivity and messenger RNA expression-based stemness index (mRNAsi) calculation

To predict responses to chemotherapy and molecular targeted drugs, we utilized the “oncoPredict” R package, which employs data from the Genomics of Drug Sensitivity in Cancer (GDSC),^7^ to assess drug responses amongst three glycolysis-lactate subtypes. We implemented a method developed by Malta et al.’s (2018) study, wherein we retained the transcriptional mRNA-based stemness index (mRNAsi) scores for LUAD patients based on pluripotent stem cells, using a one-class logistic regression machine learning algorithm (OCLR). The Wilcox test was used to determine if there was a significant difference in mRNAsi scores among the three subtypes. Additionally, we obtained 23 stemness gene sets from MsigDB and determined the stemness enrichment scores to further evaluate the differences between the glycolysis-lactate subtypes.

### Weight gene co-expression network analysis (WGCNA) to identify candidate genes

We utilized the “WGCNA” R package to construct co-expression networks of candidate genes in the GSE72094 dataset, thereby identifying representative modules of dissimilar glycolysis-lactate subtypes. The gene selection process involved discarding those with missing values, constructing a cluster tree to remove outliers, and applying network topology analysis to calculate a soft-threshold power β = 3 for a weighted adjacency matrix. We then converted this matrix into a topological overlap matrix, which enabled the production of a hierarchical cluster of genes interconnected with the corresponding dissimilarities (minModulesize = 250). Through recognizing the modules with similar patterns, an intramodular analysis was executed to quantify the Gene Significance (GS) and Module Membership (MM) of module-trait relationships. The module eigengene (ME), which represented the first principal component of each module, was then calculated, and its associations with glycolysis-lactate subtypes, TNM stage, survival information, and KRAS/EGFR/STK11/TP53 mutation were identified. We selected candidate genes based on a GS > 0.5 and MM > 0.5 threshold. Subsequently, we employed association analysis with the proteomic data from Mirhadi et al.’s study to construct a nine-quadrant map, which better visualizes the transcript and protein dynamics in the co-expression module. Proteomaps^8^ were produced to show the quantitative composition of proteomes based on Kyoto Encyclopedia of Genes and Genomes (KEGG) analyses, with each protein shown by a polygon, and polygon areas representing protein abundance.

### Prognostic glycolysis-lactate score construction and validation

We used Univariate Cox regression to detect prognostic genes in the studied module of the GSE72094 dataset. To reduce the number of filtered prognostic genes and construct a gene combination, we employed the LASSO method and Cox proportional hazard regression algorithm implemented in the “glmnet” R package. The resulting Glycolysis-lactate Score was calculated as the sum of the products of the coefficient i and RNA expression i ( ∑Coeefficienti×RNAExpressioni ). Subsequently, we validated the prognostic utility of this score in the TCGA-LUAD dataset. To confirm our findings, we also validated the score in the external GSE31210 database, which included OS and PFS information.

### Glycolysis-lactate signature on the single cell RNA (scRNA) sequencing level

We obtained a scRNA-seq dataset (GSE131907), which included 44 LUAD patients. We included 11 tumor, 11 distant normal lung, 10 normal lymph node, and 10 metastatic brain tissue samples in our study. Using the “Seurat” R package, we created a Seurat object using the raw count matrix, features, and barcodes profiles. We then removed low-quality cells based on the percentage of RNA mapped to mitochondrial genes per cell using the “PercentageFeatureSet” function. The same exclusion criteria from our previous study were applied: genes detected in fewer than three cells, fewer than 50 total detected genes, or greater than or equal to 5% mitochondrial genes (Luo et al., 2022). Nonlinear dimensionality reduction was carried out by PCA, and we identified the top 20 principal components (PCs) using the uniform manifold approximation and projection (UMAP) algorithm. Genes differentially expressed in each cluster with an adjusted *p* < 0.05 and ∣log2(Fold Change (FC))∣ > 0.5 were designated as marker genes. We annotated each cluster using the recently developed “ScType” R package, which provides unbiased and improved cell type annotations (Ianevski et al., 2022). Finally, we generated glycolysis-lactate signatures on the single-cell level using the “AddModuleScore” function and TISCH website.^9^

### Statistical analysis

All statistical analyses were performed using Rstudio software (version 4.1.1). We used the “cor.test” function in R to compute bi-directional Spearman’s correlation. To produce the survival curves and assess survival differences, we utilized the K-M plot and log-rank test. We employed the “clusterProfiler” R package to identify enriched functions associated with specific genes. For all comparisons, statistical significance was set at a two-tailed *p*-value of less than 0.05.

## Results

### Development of a scoring method based on TCGA-LUAD-microbiome

**Figure 1:**
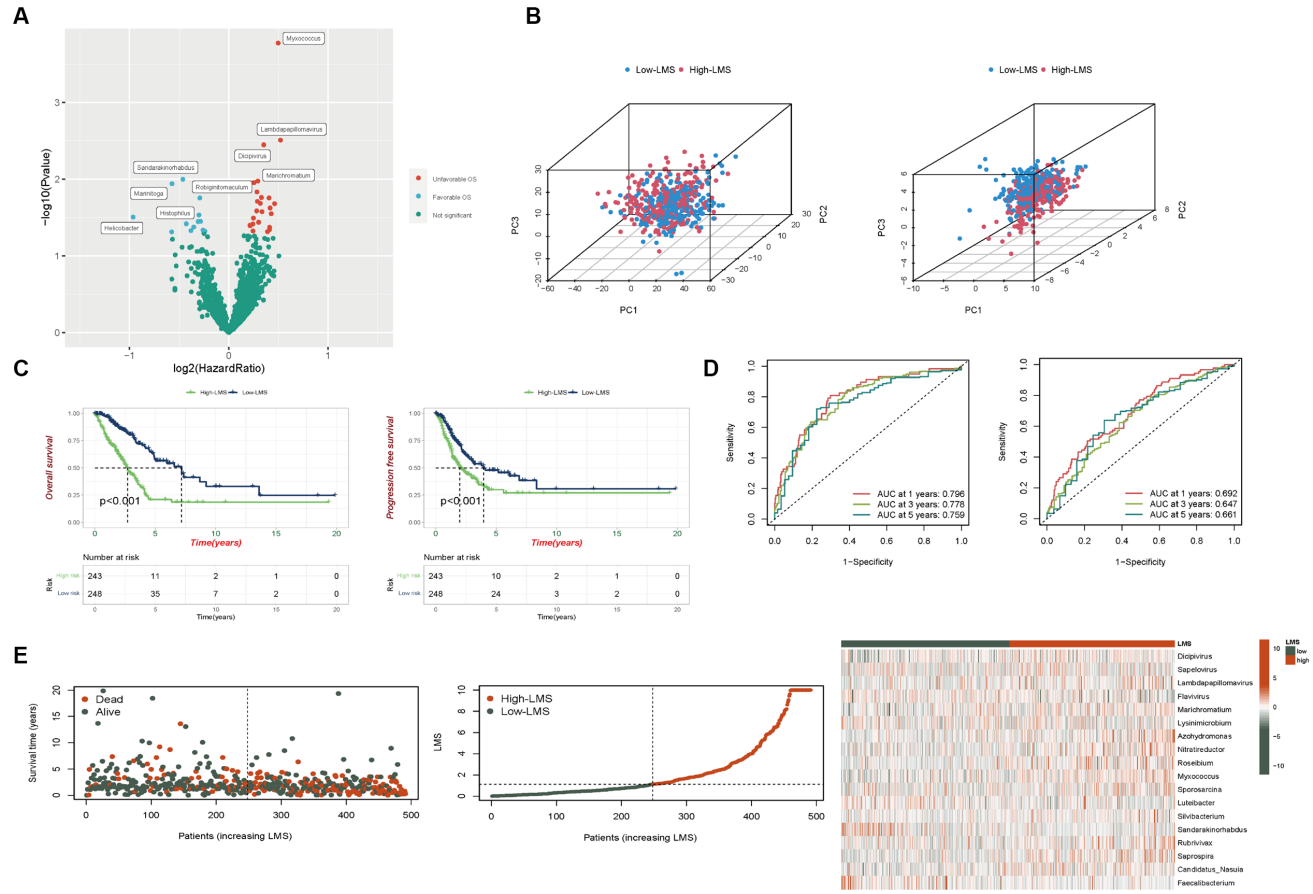
Development of a scoring method based on TCGA-LUAD-microbiome. **(A)** Volcano plot displays the relationship between microbial abundance and overall survival (OS). **(B)** Categorization of high and low LMS patients before and after LMS construction based on the PCA (Left: Number of microbes = 1,406, Right: Number of microbes = 18). **(C)** Kaplan–Meier analysis of OS and progression free survival (PFS) in total set. **(D)** Risk scores predicting OS in total set using time-independent ROC analysis. **(E)** The distribution of survival status, risk scores and abundance of 18 prognostic microbes in total set.

We have created a flowchart depicting our study process and included it as Supplementary Figure S1. The TCGA-LUAD-microbiome dataset was divided into a training set (*n* = 246) and a testing set (*n* = 245). Univariate Cox regression analysis was used to identify 38 out of 1,406 genera whose abundance was associated with prognostic value in the 246 LUAD samples from the TCGA training dataset. Of these, 14 genera, namely *Helicobacter*, *Histophilus*, *Luteibacter*, *Marinitoga*, *Sandarakinorhabdus*, *Simplexvirus*, *Stomatobaculum*, *Roseiflexus*, *Methylopila*, *Sapelovirus*, *Faecalibacterium*, *Flavivirus*, *Belnapia* and *Lysinimicrobium* correlated with favorable OS while the abundance of the remaining 24 genera was associated with worse survival (*p* < 0.05, as depicted in Figure 1A and Supplementary Figure S2A). We further investigated the correlation between clinical parameters and microbial abundance, revealing that the abundance of *Marichromatium*, *Roseibium* and *Sporosarcina* was positively correlated with increasing tumor stages whereas the abundances of *Luterbacter* and *Sandarakinorhabdus* were attenuated in the stage III-IV group (Supplementary Figure S2B). Based on the microbial abundance, patients were stratified into high and low groups, and the cut-off points that demonstrated the most significant OS differences were determined. The Kaplan–Meier curves showed that patients with high abundance of *Marichromatium*, *Roseibium* and *Sporosarcina* had notably shorter OS, while those with high abundance of *Luterbacter* and *Sandarakinorhabdus* had a more favorable prognosis (Supplementary Figure S2C). We then performed Lasso-penalized Cox regression to select potential prognostic microbes, and the shrinkage penalty parameters for Lambda (λ) were determined by ten-fold cross-validation (Supplementary Figure S2D). Multivariate Cox regression analysis was finally performed by including the 18 prognostic microbes with non-zero regression coefficients to obtain the regression coefficients for each microbe. The derived scoring method was

$$\begin{array}{l} \mathrm{LMS}=\left(0.244\times Dicipivirus\right)+\left(-0.239\times Sapelovirus\right)\\ \;\;\;\;\;\;\;\;\;\;\;\;\;\;\;\;+\left(0.473\times Lambdapapillomavirus\right)+\left(-0.179\times Flavivirus\right)\\ \;\;\;\;\;\;\;\;\;\;\;\;\;\;\;\;+\left(0.350\times Marichromatium\right)+\left(-0.234\times Lysinimicrobium\right)\\ \;\;\;\;\;\;\;\;\;\;\;\;\;\;\;\;+\left(0.399\times Azohydromonas\right)+\left(0.210\times Nitratireductor\right)\\ \;\;\;\;\;\;\;\;\;\;\;\;\;\;\;\;+\left(0.304\times Roseibium\right)+\left(0.318\times Myxococcus\right)\\ \;\;\;\;\;\;\;\;\;\;\;\;\;\;\;\;+\left(0.233\times Sporosarcina\right)+\left(-0.362\times Luteibacter\right)\\ \;\;\;\;\;\;\;\;\;\;\;\;\;\;\;\;+\left(0.417\times Silvibacterium\right)+\left(-0.930\times Sandarakinorhabdus\right)\\ \;\;\;\;\;\;\;\;\;\;\;\;\;\;\;\;+\left(0.272\times Rubrivivax\right)+\left(0.326\times Saprospira\right)\\ \;\;\;\;\;\;\;\;\;\;\;\;\;\;\;\;+\left(0.395\times Candidatus\_Nasuia\right)+\left(-0.295\times Faecalibacterium\right). \end{array}$$

### Evaluation of the performance of the LMS

LMS were calculated according to the formula and patients were divided into high (*n* = 243) and low-LMS groups (*n* = 248) based on the median LMS. In Figure 1B, we employed PCA to assess the discriminative power of the complete set of microbes (1,406 in total, left plot) and the specific set of 18 microbes used in the LMS (right plot). For the PCA incorporating all 1,406 microbes, the proportions of variance (R2) for PC1, PC2, and PC3 were 0.353, 0.035, and 0.023, respectively, with Q2 = 0.977. The *p*-values for the differences between the high and low LMS groups were < 0.001 for PC1, 0.542 for PC2, and 0.314 for PC3. For the PCA using the 18 LMS microbes, the proportions of variance (R2) for PC1, PC2, and PC3 were 0.156, 0.107, and 0.075, respectively, with Q2 = 0.954. The *p*-values for the differences between the high and low LMS groups were < 0.001 for PC1, 0.485 for PC2, and < 0.001 for PC3. This demonstrates a significant difference for both PC1 and PC3, indicating that this LMS is more effective in distinguishing between the two patient groups (Figure 1B).

In the training set, patients in the high-LMS group (*n* = 123) exhibited significantly worse prognostic outcomes compared to those in the low-LMS group (*n* = 123). Kaplan–Meier analysis indicated that the low-LMS group had longer OS and progression-free survival (PFS) times than the high-LMS group, as depicted in Supplementary Figures S3A,B. The AUC values for LMS were 0.905, 0.896, and 0.921 for OS and 0.789, 0.758, and 0.798 for PFS at years 1, 3, and 5 respectively, as illustrated in Supplementary Figures S3D,E. Furthermore, the risk plot demonstrated a remarkable increase in mortality rate with higher LMS, as presented in Supplementary Figure S3C. Distribution of survival status and microbial abundance among the 246 patients in the training cohort are presented in Supplementary Figure S3C. Next, the test set (*n* = 245) and entire dataset (*n* = 491) were used to verify the accuracy of LMS. In the testing set, survival curve analysis indicated that OS was significantly longer in the group with low levels of LMS, while PFS showed no significant difference (Supplementary Figures S3A,B). The AUC values obtained by ROC analysis for 1-,3-, and 5-years of OS and PFS were reported. The AUC values were 0.636, 0.659, and 0.596 and 0.602, 0.537 and 0.547, respectively (Supplementary Figures S3D,E). The distribution of LMS, survival status and microbiome abundance levels observed in the validation cohort were similar to those obtained in the training cohort (Supplementary Figure S3C).

In the entire dataset, we observed significant survival advantage for both OS and PFS in the group with low LMS levels (Figure 1C). ROC analysis showed that AUC values, respectively, for 1-, 3-, and 5-years were 0.796, 0.778, and 0.759 for OS and 0.692, 0.647, and 0.661 for PFS (Figure 1D). Additionally, the risk curve and microbe heatmap exhibited similar findings to the ones observed in the training and testing cohorts (Figure 1E). In summary, our results suggest that LMS could potentially serve as a prognostic biomarker for LUAD.

**Figure 2:**
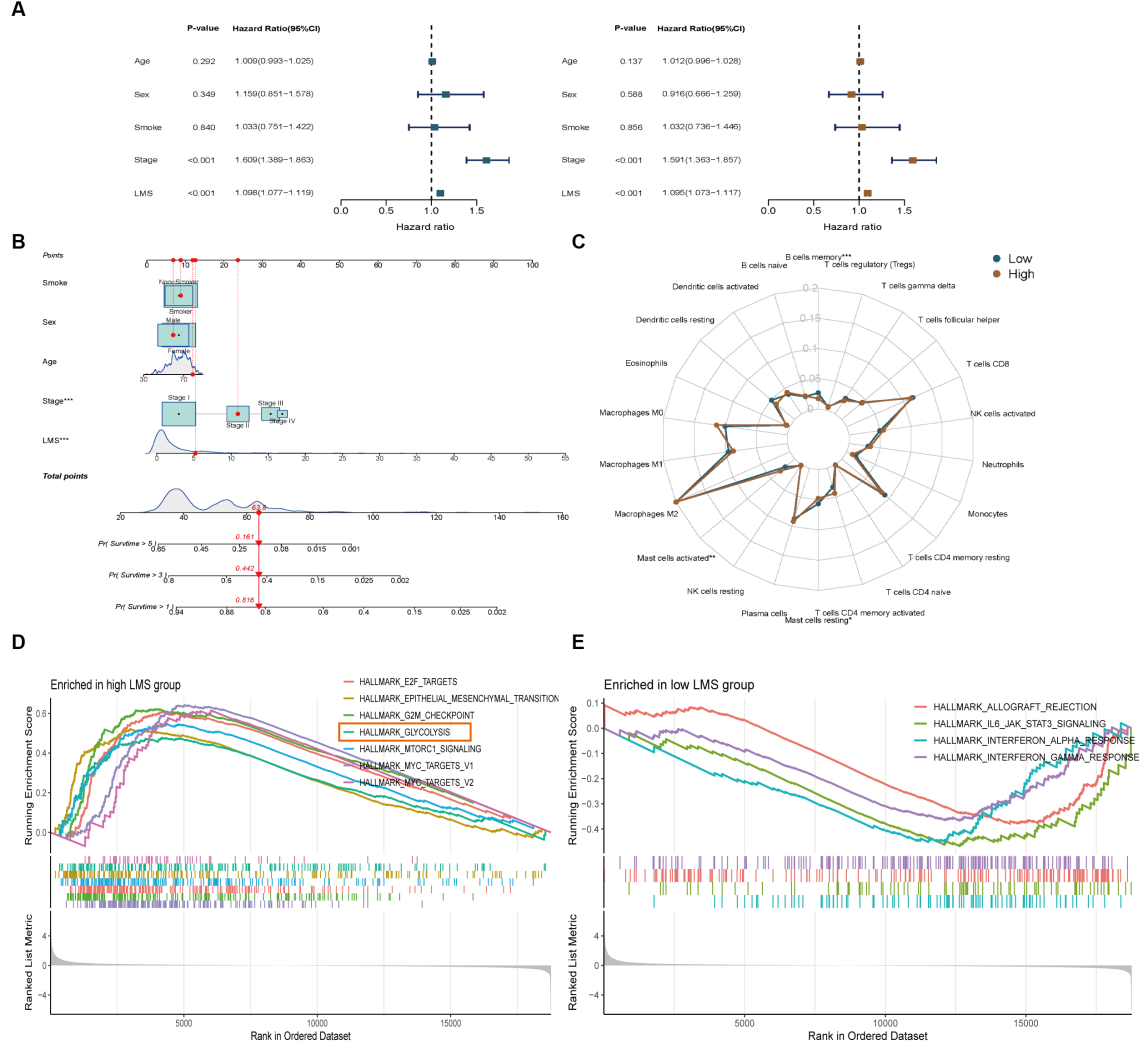
Identification of tumor infiltrating immunocytes and enrichment analysis of LMS groups. **(A)** Univariate and multivariate Cox regression analyses to verify the prognostic values of various clinicopathological factors and LMS. Brown: Univariate Cox regression; Blue: Multivariate Cox regression. **(B)** A nomogram was created that included both the LMS and clinical factors. **(C)** Radar plot showed the proportions of infiltrating immunocytes in the high and low LMS groups. **(D,E)** GSEA Hallmark enrichment analysis in the high **(D)** and low **(E)** LMS groups. ^***^ *p* < 0.001.

To evaluate the independent prognostic value of LMS, we performed both univariate and multivariate Cox regression analysis that included other clinical features. The results showed that LMS could serve as an independent predictor for OS even after adjusting for age, sex, smoking status and stage in the multivariable analysis (Figure 2A). Based on the results obtained from the multivariable Cox regression analysis, we developed a prognostic nomogram that can predict 1-, 3-and 5- year OS in a clinical setting (Figure 2B). The calibration curves of the nomogram for 1-, 3-and 5- year OS were plotted, and the prediction line was almost coincident with the best-fit line (45°C line). This result demonstrates good agreement between predicted and actual probabilities, and high precision in distinguishing most survival outcomes at these time points (Supplementary Figure S3F).

### Identification of tumor infiltrating immunocytes and enrichment analysis of LMS groups

We examined the relationship between LMS and tumor-infiltrating immune cells in LUAD. We used the CIBERSORT method to estimate the proportions of immune cells in each TCGA-LUAD transcriptome sample. The low-LMS group had a higher proportion of memory B cells and resting mast cells, while the high-LMS group showed a higher proportion of activated mast cells (Figure 2C).

We conducted GSEA Hallmark enrichment analysis to elucidate the underlying mechanisms that differentiate high-and low-LMS groups. The results showed that high-LMS group was significantly enriched in cell cycle-related pathways such as HALLMARK_E2F_TARGETS (NES = 2.13, NOM *p*-value < 0.001 and FDR *q*-value < 0.001), HALLMARK_G2M_CHECKPOINT (NES = 2.18, NOM *p*-value < 0.001 and FDR *q*-value < 0.001), HALLMARK_MTORC1_SIGNALING (NES = 1.92, NOM *p*-value < 0.001 and FDR *q*-value<0.001), HALLMARK_MYC_TARGETS_V1 (NES = 2.25, NOM *p*-value < 0.001 and FDR *q*-value < 0.001) and HALLMARK_MYC_TARGETS_V2 (NES = 1.80, NOM value of *p* = 0.001 and FDR *q*-value = 0.006) (Figure 2D). On the other hand, low-LMS group was enriched in anti-infective pathways such as HALLMARK_ALLOGRAFT_REJECTION (NES = -1.47, NOM *p*-value = 0.005 and FDR *q*-value = 0.013), HALLMARK_IL6_JAK_STAT3_SIGNALING (NES = -1.62, NOM *p*-value = 0.005 and FDR *q*-value = 0.01), HALLMARK_INTERFERON_ALPHA_RESPONSE (NES = -1.60, NOM value of *p* = 0.004 and FDR *q*-value = 0.01), HALLMARK_INTERFERON_GAMMA_RESPONSE (NES = -1.45, NOM *p*-value = 0.007 and FDR *q*-value = 0.01) (Figure 2E). Notably, HALLMARK_GLYCOLYSIS (NES = 1.67, NOM *p*-value < 0.001 and FDR *q*-value = 0.003) was significantly enriched in the high-LMS group, indicating a possible important role of glycolysis in intratumor microbe-related LUAD, which was distinct from the cell-cycle related pathways (Figure 2D).

### Immune characteristics associated with glycolysis and lactate metabolic patterns in LUAD

**Figure 3:**
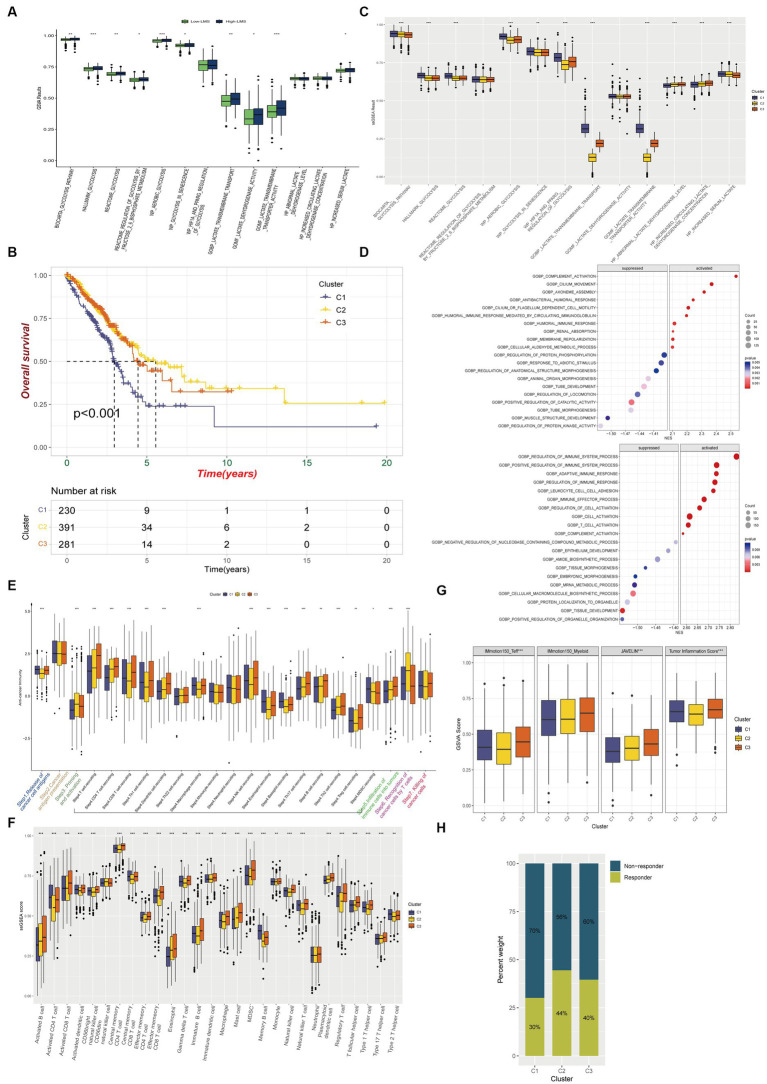
Immune characteristics among glycolysis and lactate metabolic patterns in LUAD. **(A)** Comparison of glycolysis and lactate metabolic gene sets enrichments between high and low LMS groups. **(B)** Kaplan–Meier overall survival curves for LUAD patients among different clusters. **(C)** Box plot FIGURE 3 (Continued)displays the differences of 13 ssGSEA glycolysis and lactate results among the three clusters. **(D)** Gene Ontology enrichment analysis shows biological process differences between Cluster 2 and Cluster 1 (Up) or Cluster 3 and Cluster 1(Down). **(E)** Different activation status of cancer immune cycles among three clusters. **(F)** Infiltration status of immune cells in TIME among three clusters. **(G)** Box plot shows the difference among three clusters in IMmotion150_Teff, IMmotion150_Myeloid, JAVELIN and Tumor inflammation signature. **(H)** The proportion of immunotherapy responders and non-responders in the three clusters estimated by TIDE algorithm. ^*^ *p* < 0.05, ^**^ *p* < 0.01, ^***^ *p* < 0.001.

To determine whether significant differences in glycolysis and lactate-regulated patterns existed between high-and low- LMS groups, ssGSEA was conducted using 13 glycolysis and lactate metabolic gene sets. The results showed that 10 of these 13 gene sets exhibited a higher level of enrichment in the high-LMS group, while the remaining 3 gene sets showed no significant differences (Figure 3A). We found that genes related to glycolysis and lactate metabolism are closely associated with the lung-resident microbiome and have prognostic value in LUAD. Inspired by these results, we performed hierarchical clustering on TCGA-LUAD patient data to identify three distinct groups (Cluster 1, Cluster 2, and Cluster 3) based on regulated patterns in LUAD (Supplementary Figure S4A). Patients in Cluster 1 had significantly worse survival outcomes compared to those in Clusters 2 and 3 (Figure 3B). Moreover, most glycolysis and lactate metabolic gene sets were upregulated in Cluster 1, while certain gene sets related to lactate dehydrogenase exhibited no significant differences (Figure 3C). Gene Ontology (GO) enrichment analysis revealed suppression of several immune-related biological processes, such as regulation of immune system processes, regulation of immune response, and T-cell activation, in Cluster 1, suggesting potential differences in immune status among the three clusters (Figure 3D).

We investigated whether glycolysis and lactate-regulated patterns played a role in the TIME. The seven-step cancer immunity cycle, as delineated by Chen and Mellman (2013), offers a structured understanding of the successive stages inherent in anti-tumor immune responses: Step1 Liberation of Cancer Cell Antigens: This initial phase encompasses the apoptosis and necrosis of neoplastic cells, concomitant with the extrication of antigens. Step2 Capture and Processing of Cancer Antigens: Antigen-presenting cells, notably dendritic cells, seize and process these antigens in preparation for their presentation to the immune effector cells. Step3 Presentation of Antigens: The processed antigens are exhibited to immune cells on the surface of antigen-presenting cells, facilitated by the Major Histocompatibility Complex (MHC). Step4 Priming and Activation: Recognition and binding of antigens by immune cells within lymph nodes instigate their activation and proliferation, instigating their trajectory towards the tumor site. Step5 Immune Cell Trafficking and Infiltration: Post-activation, immune cells egress from the lymph nodes, traverse the circulatory system, and subsequently infiltrate the tumor. Step6 Recognition of Cancer Cells: Within the confines of the tumor microenvironment, T cells recognize and affix to antigens expressed on the tumor cells. Step7 Elimination of Cancer Cells: T cells discharge cytotoxic molecules, culminating in the annihilation of tumor cells. This sequence engenders the release of additional antigens, thereby perpetuating the cycle. Our results showed a significant decrease in anti-cancer immunity status across the cycle in Cluster 1, compared to Clusters 2 and 3, indicating that patients in Cluster 1 may exhibit a constrained tumor immune activation and immune infiltration into the TIME (Figure 3E).

We used established gene markers to confirm that most of the tumor infiltrating lymphocytes, such as activated CD8+ T cells, dendritic cells (DCs), CD56+ natural killer cells, macrophages, and natural killer T cells, exhibited significantly higher levels in Cluster 3 and lower levels in Cluster 1 (Figure 3F). We used the ESTIMATE immune score as a surrogate for immune infiltration and found that Cluster 1 had the lowest immune score (Supplementary Figure S4B). Our findings suggested that Cluster 1 represented a non-inflamed TIME phenotype that may exhibit insensitivity to immune checkpoint blockade (ICB) therapy, while Cluster 3 represented an inflamed phenotype that may exhibit sensitivity to such therapy. The IMmotion150_Teff signature, JAVELIN Pathway, and Tumor inflammation signature from the four reported immune-related pathways exhibited significantly lower levels in Cluster 1, while most of the pathways reported by Mariathasan et al., which possessed immunotherapy efficacy predicting value, were upregulated in Cluster 1 when compared to Clusters 2 and 3 (Figure 3G; Supplementary Figure S4C). Using the Tumor Immune Dysfunction and Exclusion (TIDE) algorithm, we predicted that Cluster 1 was less responsive to immunotherapy than Clusters 2 and 3 (Figure 3H).

### Chemo and targeted therapy response and cancer stemness levels among identified clusters

**Figure 4:**
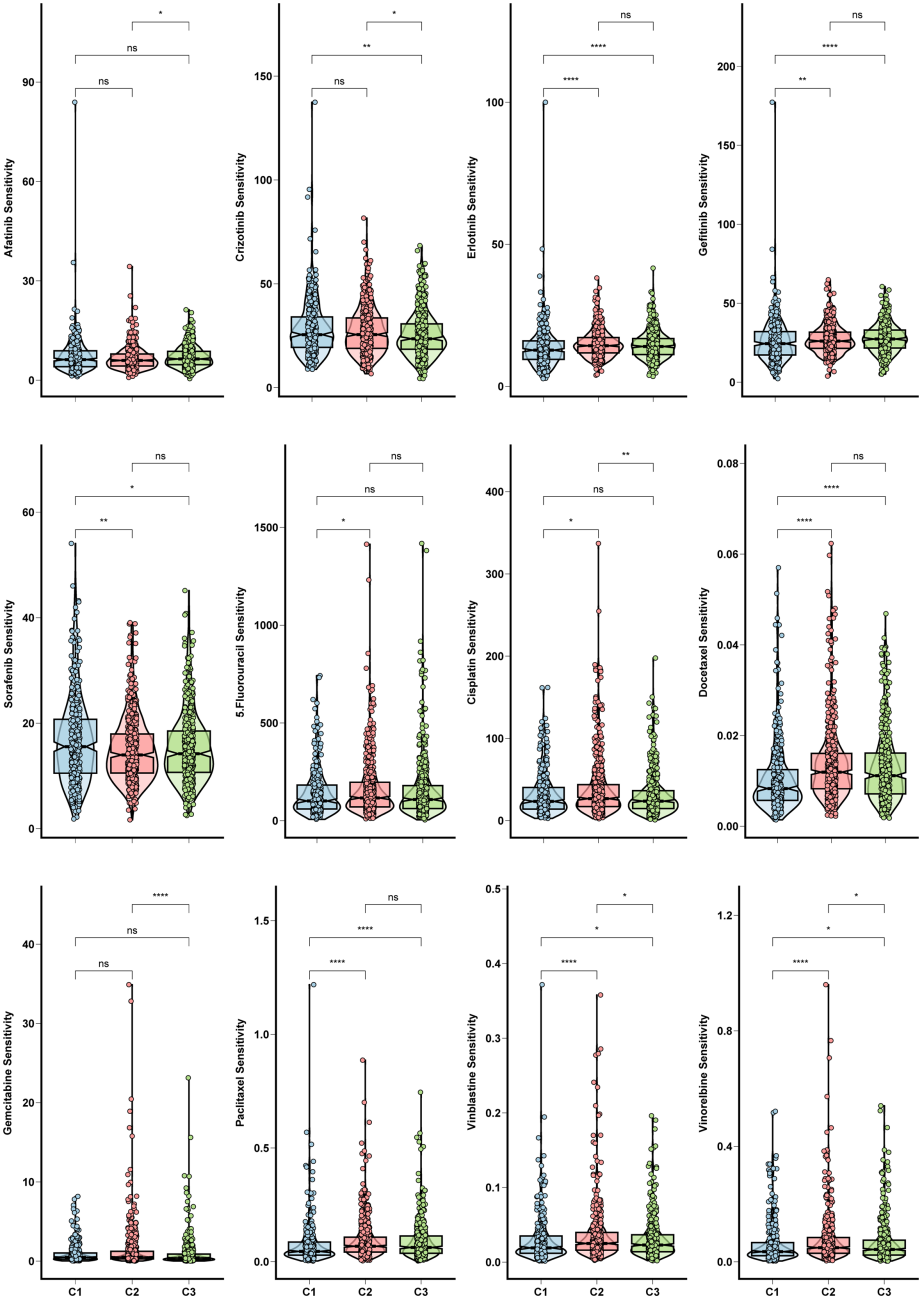
Chemo and targeted therapy response and cancer stemness among clusters. Box plots for the sensitivity of chemotherapy and target therapy drugs among three glycolysis-lactate clusters.

**Figure 5:**
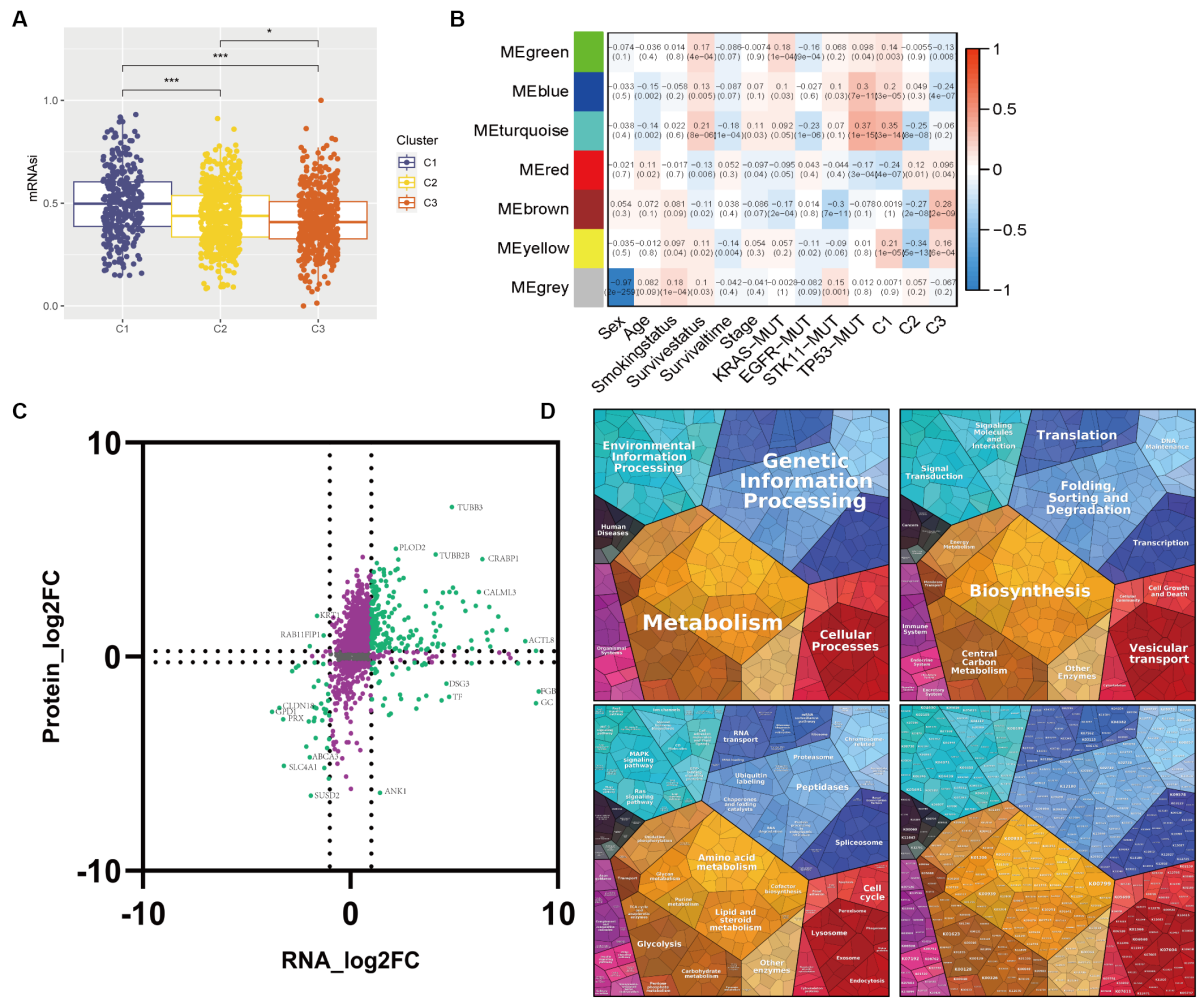
Correlations between transcriptome-proteome in the WGCNA identifying module. **(A)** Box plot of the comparison of the mRNAsi among three clusters. **(B)** Heatmap of the correlation between module eigengenes and clinicopathological characteristics as well as the three clusters. **(C)** Nine-quadrant diagram for the transcriptome-proteome correlations. The horizontal axis represents the log2 fold change of genes and the vertical axis represents the log2 fold change of proteins. **(D)** Proteomap displays the relative abundance of proteins in the module turquoise with functional cellular compartments.

Chemoresistance is a significant obstacle that affects the success of chemotherapy and targeted therapy in lung adenocarcinoma detected at an advanced stage (Du et al., 2021). We calculated *in vivo* drug sensitivity for several chemotherapy and targeted therapy drugs and compared sensitivity between glycolysis-based and lactate-based clusters. Cluster 1 had significantly reduced sensitivity to Erlotinib, Gefitinib, Docetaxel, Paclitaxel, Vinblastine, and Vinorelbine, indicating a decrease in sensitivity to these agents. Conversely, Cluster 1 exhibited higher sensitivity to Crizotinib and Sorafenib (Figure 4). The stemness of the three clusters was assessed using the OCLR algorithm. Observations of mRNAsi values indicated that Cluster 1 exhibited increased tumor stemness, whereas Cluster 3 had predominantly low values (Figure 5A). In addition, Cluster 1 showed enrichment in the majority of the 26 stemness gene sets, as shown in Supplementary Figure S3D.

### Correlations between transcriptome-proteome in the WGCNA identifying module

To identify candidate module genes, we performed WGCNA on the GSE72094 cohort in response to the unfavorable survival and immunotherapy efficacy in Cluster 1 patients (Figure 5B). Seven gene modules were identified with similar expression patterns, out of which the turquoise module appeared to have the strongest positive correlation with Cluster 1 (ME = 0.35, *p* = 3e-14) and the most negative association with Cluster 2 (ME = -0.25, *p* = 8e-08). Therefore, the turquoise module with 5,133 genes was selected as the candidate module for further analysis.

Multi-omics analysis can minimize the biological bias in single-omic data and illuminate various cellular aspects based on the distinct omics used for the study. We performed transcriptome-proteome analysis to gain a thorough understanding of molecular interactions in the candidate module. 1,633 correlation pairs were selected and illustrated in a nine-quadrant plot (Figure 5C). Most genes were assigned to the second quadrant, which indicated higher protein expression levels compared to gene expression, followed by a high percentage of genes consistent with proteins in the third quadrant. Proteomap pathway analysis was performed to classify the functions of these proteins. The results revealed that the protein changes have a significant effect on various pathways including Metabolism, Genetic Information Processing, Cellular Processes, and Environmental Information Processing with a pronounced focus on the Glycolysis pathway (Figure 5D).

### Glycolysis-lactate score as a prognostic tool for LUAD patients

In the initial step, we performed a univariable Cox regression model to identify 1,290 genes in the turquoise module with prognostic values. These genes underwent a ten-fold cross-validation LASSO method, resulting in 19 genes which were selected to constitute the glycolysis-lactate score (Supplementary Figure S4E). Using Cox proportional hazard regression, we created the glycolysis-lactate score based on the following 19 genes:

$$\begin{array}{l} \mathrm{glycolysis}-\mathrm{lactate score}=\left(0.080\times \mathrm{SLC}2\mathrm{A1}\right)+\left(-0.008\times \mathrm{SLCO}4\mathrm{C1}\right)\\ \;\;\;\;\;\;\;\;\;\;\;\;\;\;\;\;\;\;\;\;\;\;\;\;\;\;\;\;\;\;\;\;\;\;\;\;\;\;\;\;\;\;\;\;\;\;\;\;\;\;\;\;\;\;\;\;\;\;+\left(0.053\times \mathrm{EHBP1}\right)+\left(-0.072\times \mathrm{RAPGEF6}\right)\\ \;\;\;\;\;\;\;\;\;\;\;\;\;\;\;\;\;\;\;\;\;\;\;\;\;\;\;\;\;\;\;\;\;\;\;\;\;\;\;\;\;\;\;\;\;\;\;\;\;\;\;\;\;\;\;\;\;\;+\left(-0.010\times \mathrm{SLC}2A13\right)+\left(-0.062\times \mathrm{RPS}6\mathrm{KA3}\right)\\ \;\;\;\;\;\;\;\;\;\;\;\;\;\;\;\;\;\;\;\;\;\;\;\;\;\;\;\;\;\;\;\;\;\;\;\;\;\;\;\;\;\;\;\;\;\;\;\;\;\;\;\;\;\;\;\;\;\;+\left(-0.004\times \mathrm{BBS5}\right)+\left(0.013\times \mathrm{GJB3}\right)\\ \;\;\;\;\;\;\;\;\;\;\;\;\;\;\;\;\;\;\;\;\;\;\;\;\;\;\;\;\;\;\;\;\;\;\;\;\;\;\;\;\;\;\;\;\;\;\;\;\;\;\;\;\;\;\;\;\;\;+\left(-0.065\times \mathrm{EIF}4\mathrm{E3}\right)+\left(-0.041\times \mathrm{IRX2}\right)\\ \;\;\;\;\;\;\;\;\;\;\;\;\;\;\;\;\;\;\;\;\;\;\;\;\;\;\;\;\;\;\;\;\;\;\;\;\;\;\;\;\;\;\;\;\;\;\;\;\;\;\;\;\;\;\;\;\;\;+\left(-0.080\times \mathrm{ZNF}319\right)+\left(-0.222\times \mathrm{MLLT1}\right)\\ \;\;\;\;\;\;\;\;\;\;\;\;\;\;\;\;\;\;\;\;\;\;\;\;\;\;\;\;\;\;\;\;\;\;\;\;\;\;\;\;\;\;\;\;\;\;\;\;\;\;\;\;\;\;\;\;\;\;+\left(-0.010\times \mathrm{SLC}2\mathrm{6A}11\right)+\left(-0.335\times \mathrm{ASTE1}\right)\\ \;\;\;\;\;\;\;\;\;\;\;\;\;\;\;\;\;\;\;\;\;\;\;\;\;\;\;\;\;\;\;\;\;\;\;\;\;\;\;\;\;\;\;\;\;\;\;\;\;\;\;\;\;\;\;\;\;\;+\left(-0.060\times \mathrm{CCDC}2\mathrm{8A}\right)+\left(-0.027\times \mathrm{ISCU}\right)\\ \;\;\;\;\;\;\;\;\;\;\;\;\;\;\;\;\;\;\;\;\;\;\;\;\;\;\;\;\;\;\;\;\;\;\;\;\;\;\;\;\;\;\;\;\;\;\;\;\;\;\;\;\;\;\;\;\;\;+\left(0.059\times \mathrm{ASCC1}\right)+\left(0.008\times \mathrm{RHOV}\right)\\ \;\;\;\;\;\;\;\;\;\;\;\;\;\;\;\;\;\;\;\;\;\;\;\;\;\;\;\;\;\;\;\;\;\;\;\;\;\;\;\;\;\;\;\;\;\;\;\;\;\;\;\;\;\;\;\;\;\;+\left(-0.071\times \mathrm{LIPT1}\right). \end{array}$$

**Figure 6:**
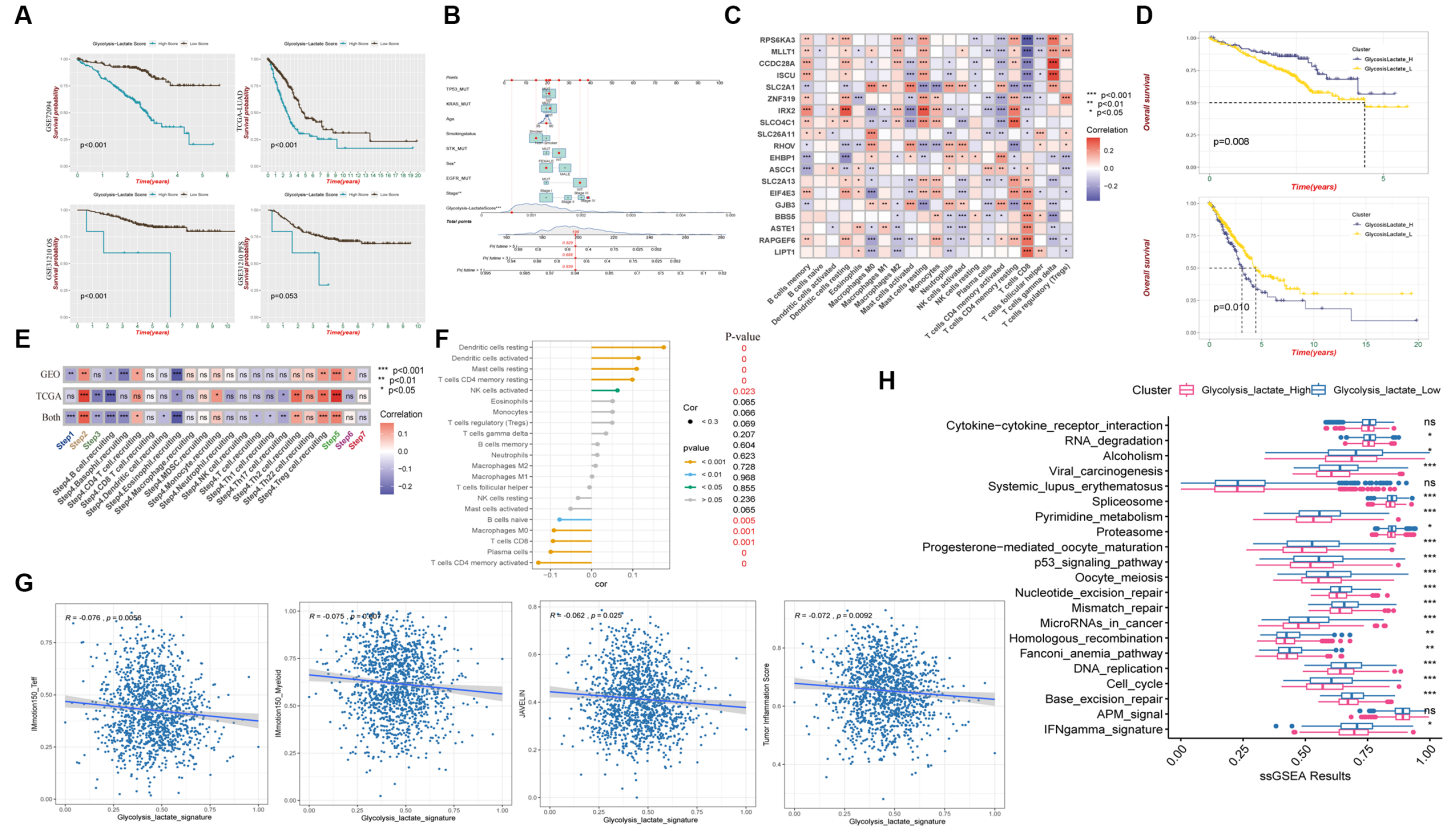
Glycolysis-lactate score construction and clinical significance as well as immune characteristic. **(A)** Kaplan–Meier analysis shows overall survival differences between high and low glycolysis-lactate score in GSE72094, TCGA-LUAD and GSE31210 cohorts and progression free survival differences in GSE31210 cohort. **(B)** A nomogram was created that included both the glycolysis-lactate score and clinical factors. **(C)** Correlation heatmap between glycolysis-lactate gene signature and infiltrating immune cells in GSE72094 and TCGA-LUAD. **(D)** Kaplan–Meier analysis shows overall survival differences between glycolysislactate_high and glycolysislactate _low signature groups in GSE72094 (Up) and TCGA-LUAD (Down). **(E)** Correlation heatmap between glycolysis-lactate gene signature and cancer immunity cycles in the GSE72094 and TCGA-LUAD, respectively. **(F)** Lollipop diagram was generated to show the association between immune cells and glycolysis-lactate gene signature. **(G)** Correlation between glycolysis-lactate signature and IMmotion150_Teff, IMmotion150_Myeloid, JAVELIN and Tumor inflammation signature, respectively. **(H)** Enrichment of each immunotherapy related pathways between high and low glycolysis-lactate signature groups. ^*^ p < 0.05, ^**^ *p* < 0.01, ^***^ *p* < 0.001, ns not significant.

We observed that patients with a higher glycolysis-lactate score exhibited significantly unfavorable survival in the GSE72094 training cohort (Figure 6A). Importantly, we validated the glycolysis-lactate score in the external TCGA-LUAD and GSE31210 cohort, high glycolysis-lactate score group exhibited inferior overall survival outcome in both cohorts. Notably, our findings indicate that the glycolysis-lactate score demonstrated negative predictive value for progression free survival in GSE31210 cohort. We also imported information on patients’ age, sex, smoking status, tumor stage, TP53/KRAS/STK/EGFR mutation status, and risk score to construct a nomogram that demonstrated a high prognostic value for 1-, 3-, and 5-year survival (Figure 6B). The ROC analysis showed that the 1-, 3-, and 5-year AUC values are 0.749, 0.790, and 0.829 for OS (Supplementary Figures S4F,G). Our results suggest that glycolysis-lactate score could serve as a valuable prognostic tool for individual LUAD patients.

### Using glycolysis-lactate signature to predict immunophenotyping and immunotherapy efficacy

To evaluate the immune characteristics of LUAD patients accurately, we conducted a correlation analysis of the 19 genes in our glycolysis-lactate score and immune infiltrating cells. As a result of analyzing GSE72094 and TCGA-LUAD cohorts, we found that SLC2A1 and IRX2 exhibited correlations with most immune infiltrating cells (Figure 6C). CD8+ T cells manifested significant correlations with 17 genes in the glycolysis-lactate score, excluding RHOV and EHBP1. Interestingly, we discovered that most of the 19 genes correlated with the infiltration of memory resting CD4+ T cells, memory activated CD4+ T cells, and resting dendritic cells in the GSE72094 cohort but not in CD8+ T cells (Supplementary Figures S5A,B). We hypothesized that these 19 genes could serve as a glycolysis-lactate gene signature to predict immune characteristics. Thus, we adopted the ssGSEA algorithm to construct a glycolysis-lactate gene signature in the TCGA-GEO combined dataset, where 1,308 samples were classified into the glycolysislactate_high and glycolysislactate_low groups. Survival analysis showed that the glycolysislactate_high group presented poor survival outcomes in the TCGA-LUAD cohort while indicating an extended survival in the GSE72094 cohort (Figure 6D).

A Tracking Tumor immunophenotype analysis was conducted to demonstrate the relationship between cancer cell cycles and glycolysis-lactate gene signature. Cancer antigen presentation, regulatory T cells (Tregs) recruiting, infiltration of immune cells into tumors showed significant positive correlations whereas B cell and Eosinophil recruiting were negatively associated with glycolysis-lactate signature both in TCGA-LUAD and GSE72094 cohorts (Figure 6E). Positive correlations were observed between the glycolysis-lactate signature and the infiltration of tumor lymphocytes such as resting and activated dendritic cells, resting mast cells, memory resting CD4+ T cells and activated NK cells, while memory activated CD4+ T cells, plasma cells, CD8+ T cells, Macrophages M0 and naïve B cells were negatively correlated (Figure 6F). Moreover, we conducted an immunotherapy efficacy prediction, and our signature was significantly negatively correlated with IMmotion150_Teff (*R* = −0.076, *p* = 0.0056), IMmotion150_Myeloid (*R* = −0.075, *p* = 0.007), JAVELIN signature (*R* = −0.062, *p* = 0.025) and Tumor Inflammation Score (*R* = −0.072, *p* = 0.0092) (Figure 6G). The glycolysislactate_low group showed significant upregulation of most 21 pathways associated with ICB treatment efficacy, except cytokine-cytokine receptor interaction, systemic lupus erythematosus and APM signal pathways (Figure 6H). In conclusion, the glycolysis-lactate signature constructed by us could be used to predict the immune infiltration and efficacy of ICB therapy in LUAD patients.

### Elevated glycolysis-lactate signature in LUAD epithelial cells and metastatic tissues at single-cell level

**Figure 7:**
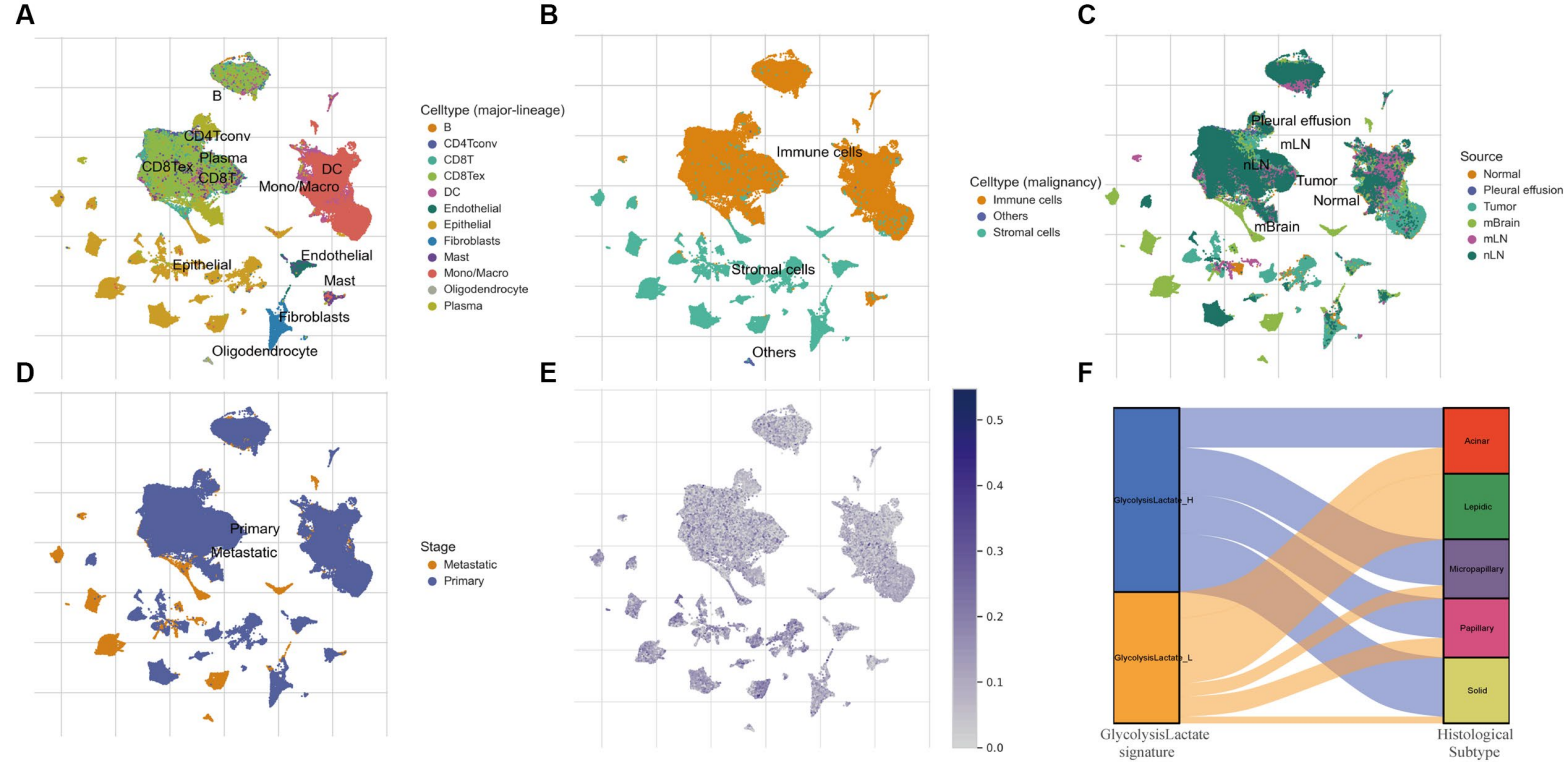
The role of glycolysis-lactate signature on the single cell level and its association with histological subtypes. **(A–D)** UMAP plots of GSE131907 and each cluster was marked by different cell types **(A,B)**, sample sources **(C)** and tumor stages **(D)**. **(E)** Distribution of glycolysis-lactate signature on the single cell level. **(F)** A Sankey diagram was illustrated to visualize the relationship between glycolysis-lactate signature and histological subtypes using the GSE58772 dataset.

We further verified our glycolysis-lactate signature on the single-cell level using the classic LUAD scRNA-seq data (GSE131907). 58 LUAD samples were annotated into B cells, conventional CD4 T cells, CD8+ T cells, exhausted CD8+ T cells, DC cells, Endothelial cells, Epithelial cells, Fibroblasts cells, Mast cells, Monocytes/Macrophages, Oligodendrocytes and Plasma cells as indicated in Figure 7A. Glycolysis-lactate signatures were particularly elevated in epithelial cells, especially in the tumor mass and brain metastasis tissue (Figures 7B,C). The metastatic tissue exhibited a significantly higher level than primary lung tumor tissue (Figure 7D).

### Glycolysis-lactate gene signature predicts histological subtypes of LUAD

LUAD is characterized by cells of various histological subtypes, exemplifying intratumor heterogeneity. Subtypes such as the lepidic are generally associated with a favorable prognosis, whereas the solid and micropapillay subtypes are linked to poorer prognostic outcomes. To elucidate the relationship between these histological subtypes and our glycolysis-lactate signature, we first employed TCGA-LUAD RNA-seq data for internal validation. The results showed that the proportion of solid and micropapillary subtypes was higher in the glycolysis-lactate-high group compared to the glycolysis-lactate-low group (Supplementary Figure S6). This initial finding prompted us to further investigate this association using an external dataset. We then utilized the GSE58772 dataset, which uniquely features subtype classification of LUAD pathology, serving as our external validation. Consistent with our findings from TCGA and previous studies, we found that all patients with the lepidic subtype were classified into the glycolysis-lactate-low group. Additionally, a trend was observed that the proportion of patients in the glycolysis-lactate-high group increased with the severity of LUAD histological subtypes (Figure 7F). These pattern implies that our glycolysis-lactate signature can effectively predict the histological subtypes of LUAD.

## Discussion

The microbiome inhabits various parts of the human body, including the gut, skin, and oral cavity, and modulates host immunity, metabolism, inflammation, and disease susceptibility (Dominguez-Bello et al., 2019). The literature offers conflicting reports regarding changes in the lung microbiome composition and diversity in lung cancer patients. In a meta-analysis by Najafi et al., the relative abundance of *Actinobacteria phylum*, *Corynebacteriaceae* and *Halomonadaceae* families, and *Corynebacterium*, *Lachnoanaerobaculum,* and *Halomonas* genera were significantly lower in lung tumor tissues (Najafi et al., 2021).

Our LMS presents unique findings regarding the potential prognostic value of the cancer resident microbiome, which can serve as a strong foundation for future investigations into the use of microbiomes as cancer biomarkers. Among the 18 microbes identified in our LMS, *Lambdapapillomavirus* was found to belong to the *Papillomaviridae* family, which has been highly correlated with cervical cancer in women (Burd and Dean, 2016). Our results also indicate that high abundance of *Lambdapapillomavirus* is an unfavorable prognostic factor for patients with lung adenocarcinoma. *Faecalibacterium* is a genus of bacteria that primarily exists in the human gut, and its sole known species, *Faecalibacterium prausnitzii*, is an important commensal bacterium (Lopez-Siles et al., 2017). In line with our findings that the abundance of *Faecalibacterium* is a favorable factor for cancer patients, Ma et al. found that *Faecalibacterium prausnitzii* inhibits the secretion of IL-6 and the phosphorylation of JAK2 in breast cancer, subsequently suppressing the progression of breast cancer cells (Ma et al., 2020). The microbiome plays a crucial role in cancer development and responses to therapy by producing metabolites, such as short-chain fatty acids, which affect epigenetic regulation, immune cell differentiation and function, angiogenesis, and apoptosis (Hou et al., 2022). For example, certain bacteria can confer resistance to chemotherapy, metabolizing drugs into inactive forms or activating drug-detoxifying enzymes, thus decreasing the efficacy of chemotherapy (Weersma et al., 2020). Altering the microbiome can potentially enhance the anti-tumor immune response by changing immune cell metabolism or function. Sivan et al. (2015) showed that *Bifidobacterium* and *B fragilis* can enhance the efficacy of checkpoint blockade immunotherapy in mouse models, indicating promising prospects for combining immunotherapy with microbiome-targeted therapy to overcome resistance mechanisms or enhance clinical efficacy.

Glycolysis is one of the most common metabolic pathways of reprogramming, that converts glucose into pyruvate and lactate, generating ATP and NADH as energy sources under aerobic or anaerobic conditions (Ganapathy-Kanniappan and Geschwind, 2013). Many cancer cells enhance glycolysis, allowing them to adapt to hypoxic and nutrient-poor conditions and evade immune surveillance (Lannering et al., 1988). This aberrant glycolysis in tumors can increase lactate production that alters the pH of the tumor microenvironment, affecting both cancer cells and immune cells (Vaupel and Multhoff, 2021). One of the mechanisms by which glycolysis influences lung cancer is through its effect on hypoxia-inducible factor 1 (HIF-1), which is activated by low oxygen levels and high glycolytic activity in tumor cells, leading to increased expression of vascular endothelial growth factor (VEGF), matrix metalloproteinases (MMPs), and glucose transporters (GLUTs). HIF-1 also suppresses the anti-tumor immune response by inducing Tregs, myeloid-derived suppressor cells (MDSCs), and immunosuppressive cytokines such as IL-10 and transforming growth factor-beta (TGF-beta) (Corzo et al., 2010; Xu Y. R. et al., 2022). Therefore, targeting glycolysis or its pathway regulators may enhance anti-tumor immunity by reducing immunosuppression or increasing the immunogenicity of cancer cells (Vaupel and Multhoff, 2021).

The reciprocal association between glycolysis and the microbiome in cancer is complex. Glycolysis can influence the composition and function of the microbiome by altering glucose availability, creating an acidic environment that may favor certain microbial species over others (Wang et al., 2019). Conversely, the microbiome can influence glycolysis by producing SCFAs or indole derivatives that can enter cancer cells *via* transporters or receptors, regulating critical enzymes or signaling pathways involved in glycolytic regulation. Studies on metabolic modeling of host–microbe interactions have shown that anaerobic microorganisms perform glycolysis in carbohydrate metabolism (Bhalla et al., 2022). Understanding how glycolysis and the microbiome interact in cancer may provide novel insights into tumor biology and immunology, as well as new therapeutic targets or strategies to improve cancer treatment outcomes.

To fully comprehend our glycolysis-lactate signature, it is imperative to delve into the biological roles of the 19 selected genes. SLC2A1 encodes a glucose transporter protein, GLUT1, which plays a key role in the uptake of glucose by cells, transporting glucose from outside the cell to the inside, thereby providing raw materials for glycolysis (Liu et al., 2022). The study by Xu Y. et al. (2022) suggests that miRNA-199a-5p promotes the malignant progression of non-small cell lung cancer by targeting SLC2A1. SLCO4C1 encodes a member of the Organic Anion Transporting Polypeptides (OATPs) family, primarily expressed on the basolateral side of renal tubular epithelial cells, responsible for transporting substances from the cell into the blood. Studies have shown that SLCO4C1 promotes the metastasis and invasion of endometrial cancer, influences the epithelial-mesenchymal transition (EMT) phenotype of endometrial cancer cells, and regulates the expression of EMT-related proteins (Hu et al., 2020). EHBP1 is an adaptor protein that regulates vesicle transport by recruiting members of the Rab8 family and Eps15 homology domain-containing proteins 1/2 (EHD1/2) (Rai et al., 2020). It also connects the endoplasmic reticulum to the actin cytoskeleton. Research indicates that EHBP1 plays a role in regulating the transport of Na + -K + -ATPase from the Golgi to the basal membrane in the retina of Drosophila (Nakamura et al., 2020). In tumor research, an EHBP1-MET fusion was found in a patient with intrahepatic cholangiocarcinoma, increasing sensitivity to crizotinib (Yu et al., 2020). RAPGEF6 encodes a guanine nucleotide exchange factor that plays a crucial role in cell signaling. By activating Rap small GTPases, it influences a variety of cell functions, including cell proliferation, migration, and cytoskeleton remodeling (Maeta et al., 2018). A study by Lindholm et al. (2019) found that RAPGEF6 is regulated by miR-342-5p in HER2^+^ breast cancer cells, and high RAPGEF6 expression levels are closely associated with better survival. SLC2A13 is a H^+^-myo-inositol transporter, which is intimately related to sugar metabolism. A study by Le et al. (2021) found that metformin could restore the expression of the SLC2A13 gene, which is related to improved insulin sensitivity and obesity. It was also found that SLC2A13 expression was induced in human breast adenocarcinoma MCF7 cells after serum starvation, making it a potential marker for various cancer stem cells (Lee et al., 2011). RPS6KA3 is a member of the mitogen-activated protein kinase family, which responds to growth factors and other cell signals to promote cell growth and proliferation. A study by Guo and Kong (2021) revealed that RPS6KA3 plays a significant role in breast cancer proliferation, migration, and invasion. BBS5, which is part of the BBSome complex, plays a key role in protein transport within cilia, allowing the cilia to function as a sensory and signaling center for cells (Bales et al., 2020). Next, GJB3, which encodes a gap junction protein, enables the exchange of substances between cells, including metabolites and secondary messengers. Studies have shown that the expression of GJB3 significantly increases in liver metastasis of pancreatic ductal adenocarcinoma. Interestingly, GJB3 can form channels between PDAC tumor cells and accumulated neutrophils, transferring cyclic adenosine monophosphate (cAMP) from cancer cells to neutrophils, thus supporting their survival and polarization (Huo et al., 2022). Another gene of interest is EIF4E3, which recognizes and binds to the m7G cap structure at the 5′ end of mRNA, playing a crucial role in mRNA expression. Compared with normal tissues, the expression of EIF4E3 is lower in squamous cell carcinoma of the head and neck. Overexpression of EIF4E3 can induce the expression of CCL4/CCL5, playing a significant role in monocyte differentiation in the tumor microenvironment (Xu et al., 2023). Moreover, the protein encoded by IRX2 regulates the determination of cell fate, especially in the development of the nervous system. IRX2 plays a significant role in lymph node metastasis of breast cancer, and its expression significantly increases in lymph node metastasis of breast cancer (Werner et al., 2015). ZNF319, a member of the Zinc Finger Protein (ZNF) family, exhibits low expression in tumor tissues of breast cancer patients. Remarkably, in almost all subtypes of breast cancer, high expression of ZNF319 is associated with better clinical prognosis (Wang L. et al., 2022). Continuing with the discussion, MLLT1 is an acetyl/acyl-dependent epigenetic reader domain, the dysfunction of which has been implicated in the development of some aggressive cancers. In cancer stem cells of glioblastoma (GBM), there is a potentially harmful frameshift mutation in the MLLT1 gene, which occurs only in cancer stem cell samples derived from peritumoral tissues (Marei et al., 2022). SLC26A11 plays a key role in intracellular ion balance and fluid acid–base balance. In chronic myeloid leukemia (CML), there is a fusion between the SLC26A11 gene and RNF213 gene. This fusion may disrupt certain specific structural domains of the SLC26A11 protein, potentially affecting its normal sulfate transport function (Zhou et al., 2013). The protein encoded by ASTE1 is thought to play a role in epigenetic signaling. Research suggests that mutations in ASTE1 are induced by the Epstein–Barr virus in gastric mucosal cells, leading to the autonomous expression of CXCL9 by cancer cells through the NF-κB pathway, increasing IFN-γ in the microenvironment and stimulating immune response. The expression of the CCDC28A gene changes in low-grade and high-grade gliomas, which may be related to the development of these diseases (Gahoi et al., 2020). A recent study also found that the CCDC28A gene is a significant prognostic marker for patients with colon cancer (Zhou et al., 2022). The gene ISCU encodes a protein that plays a key role in the biosynthesis of iron–sulfur (Fe-S) clusters. In diabetic nephropathy (DN), the ISCU gene may influence the level of aerobic glycolysis, which may be related to the development of the disease (Montealegre et al., 2022). A study by Xu et al. (2021) found that Quercetin can inhibit aerobic glycolysis in diabetic nephropathy by regulating the HIF-1α/miR-210/ISCU/FeS pathway, thereby combating the development of diabetic nephropathy. ASCC1 may affect cellular DNA repair mechanisms, and its germline mutation may be associated with malignant progression of Barrett’s esophagus and esophageal adenocarcinoma (Orloff et al., 2011). The RHOV gene encodes a protein that may play a crucial role in the development and metastasis of lung cancer. The expression of the RHOV gene increases in the NSCLC (Wang R. et al., 2022). Zhang et al. (2021) suggested that the RHOV gene might influence the progression of lung adenocarcinoma by activating the JNK/c-Jun signaling pathway. Additionally, a study by Chen et al. (2021) indicated that increased expression of RHOV in lung adenocarcinoma could be related to disease progression and resistance to EGFR-TKI treatment. In Leigh’s disease, mutations in the LIPT1 gene may lead to defects in pyruvate dehydrogenase (PDH) and α-ketoglutarate dehydrogenase (α-KGDH), which could be related to disease progression (Tort et al., 2014). Moreover, another study found that increased expression of LIPT1 in hepatocellular carcinoma could potentially serve as a new therapeutic target for the disease (Yan et al., 2022). Through a thorough understanding of the functions of the genes in the glycolysis-lactate signature, we found that only SLC2A1, SLC2A13, and ISCU have been previously studied for their roles in glycolysis and glucose metabolism. Although the proteins encoded by the other genes are not specifically involved in glucose metabolism, they all participate in basic cellular metabolic functions, such as ion transport, protein transport within cilia, and sulfate transport. It’s noteworthy that all 19 genes have been found to be involved in the progression of various tumors, performing unique functions. This suggests that in our future research, we should pay close attention to the functions of these genes. Our study has some limitations. Firstly, the potential variation in microbiota composition due to geographical and demographic differences, which might impact the validity of the 18-microbe prognostic score, is not addressed in our study. Most current studies on the role of microbiota in lung cancer focus on gut microbiota, and intratumoral microbiome studies are relatively scarce. Furthermore, at this stage, we were unable to obtain independent datasets with sufficient sample size from different regions for validation, which might affect the generalizability of our findings. Secondly, the findings in the proteomics and transcriptomics were not from the same sample, which may have influenced the reliability of the results to some extent. Thirdly, due to the lack of a prospective study, we only generated our results from retrospective data. Lastly, tumor immunotherapy consists of complex microorganisms, immune cells, and glycolysis signaling pathways, which indicates the role of microbiomes and glycolysis requires further validation both *in vivo* and *in vitro*.

## Conclusion

In summary, our study demonstrated that an 18-microbe prognostic score and a 19-gene glycolysis-lactate signature are predictors of the prognosis of patients with lung adenocarcinoma. The LMS, glycolysis-lactate score, and glycolysis-lactate signature have the potential to serve as predictors of immunotherapy efficacy and histological subtype, providing valuable information for precision therapy.

## Data availability statement

The original contributions presented in the study are included in the article/Supplementary material, further inquiries can be directed to the corresponding authors.

## Author contributions

XD, XC, and JQ designed the study and contributed to the study materials and consumables. YL, XD, and JQ conducted the study. YL, XC, and XD collected data. XD, XC, and YL performed the statistical analyses and interpreted the data. XD, YL, JQ, and LC wrote the manuscript. All authors contributed to the article and approved the submitted version.

## Funding

This work was supported by the National Natural Science Foundation of China (grant No. 81972175), the Major Program of Science and Technology Foundation of Jiangsu Province (No.BE2018746), and the Program of Jiangsu Medical Innovation Team (No. CXTDA2017006).

## Conflict of interest

The authors declare that the research was conducted in the absence of any commercial or financial relationships that could be construed as a potential conflict of interest.

## Publisher’s note

All claims expressed in this article are solely those of the authors and do not necessarily represent those of their affiliated organizations, or those of the publisher, the editors and the reviewers. Any product that may be evaluated in this article, or claim that may be made by its manufacturer, is not guaranteed or endorsed by the publisher.

## Footnotes

^1^https://www.cbioportal.org/

^2^https://www.ncbi.nlm.nih.gov/geo/

^3^https://cibersort.stanford.edu

^4^http://www.gsea-msigdb.org/

^5^http://biocc.hrbmu.edu.cn/TIP/

^6^http://tide.dfci.harvard.edu/

^7^https://www.cancerrxgene.org/

^8^https://proteomaps.net/

^9^http://tisch.comp-genomics.org

## References

[ref1] Atarashi K. Tanoue T. Ando M. Kamada N. Nagano Y. Narushima S.. (2015). Th17 cell induction by adhesion of microbes to intestinal epithelial cells. Cells 163, 367–380. doi: 10.1016/j.cell.2015.08.058, PMID: 26411289 PMC4765954

[ref2] Bales K. L. Bentley M. R. Croyle M. J. Kesterson R. A. Yoder B. K. Gross A. K. (2020). BBSome component BBS5 is required for cone photoreceptor protein trafficking and outer segment maintenance. Invest. Ophthalmol. Vis. Sci. 61:17. doi: 10.1167/iovs.61.10.17, PMID: 32776140 PMC7441369

[ref3] Bhalla P. Rengaswamy R. Karunagaran D. Suraishkumar G. K. Sahoo S. (2022). Metabolic modeling of host-microbe interactions for therapeutics in colorectal cancer. NPJ Syst. Biol. Appl. 8:1. doi: 10.1038/s41540-021-00210-9, PMID: 35046399 PMC8770697

[ref4] Botticelli A. Vernocchi P. Marini F. Quagliariello A. Cerbelli B. Reddel S.. (2020). Gut metabolomics profiling of non-small cell lung cancer (NSCLC) patients under immunotherapy treatment. J. Transl. Med. 18:49. doi: 10.1186/s12967-020-02231-0, PMID: 32014010 PMC6998840

[ref5] Braun D. A. Hou Y. Bakouny Z. Ficial M. Sant' Angelo M. Forman J.. (2020). Interplay of somatic alterations and immune infiltration modulates response to PD-1 blockade in advanced clear cell renal cell carcinoma. Nat. Med. 26, 909–918. doi: 10.1038/s41591-020-0839-y, PMID: 32472114 PMC7499153

[ref6] Burd E. M. Dean C. L. (2016). Human Papillomavirus. Microbiol. Spectr. 4. doi: 10.1128/microbiolspec.DMIH2-0001-2015, PMID: 27726787

[ref7] Burkitt M. D. Duckworth C. A. Williams J. M. Pritchard D. M. (2017). *Helicobacter pylori*-induced gastric pathology: insights from in vivo and ex vivo models. Dis. Model. Mech. 10, 89–104. doi: 10.1242/dmm.027649, PMID: 28151409 PMC5312008

[ref8] Charlson E. S. Bittinger K. Haas A. R. Fitzgerald A. S. Frank I. Yadav A.. (2011). Topographical continuity of bacterial populations in the healthy human respiratory tract. Am. J. Respir. Crit. Care Med. 184, 957–963. doi: 10.1164/rccm.201104-0655OC, PMID: 21680950 PMC3208663

[ref9] Charoentong P. Finotello F. Angelova M. Mayer C. Efremova M. Rieder D.. (2017). Pan-cancer Immunogenomic analyses reveal genotype-Immunophenotype relationships and predictors of response to checkpoint blockade. Cell Rep. 18, 248–262. doi: 10.1016/j.celrep.2016.12.019, PMID: 28052254

[ref10] Chen K. Y. Hsiao C. F. Chang G. C. Tsai Y. H. Su W. C. Chen Y. M.. (2015). Estrogen receptor gene polymorphisms and lung adenocarcinoma risk in never-smoking women. J. Thorac. Oncol. 10, 1413–1420. doi: 10.1097/JTO.0000000000000646, PMID: 26301798

[ref11] Chen D. S. Mellman I. (2013). Oncology meets immunology: the cancer-immunity cycle. Immunity 39, 1–10. doi: 10.1016/j.immuni.2013.07.012 23890059

[ref12] Chen H. Xia R. Jiang L. Zhou Y. Xu H. Peng W.. (2021). Overexpression of RhoV promotes the progression and EGFR-TKI resistance of lung adenocarcinoma. Front. Oncol. 11:619013. doi: 10.3389/fonc.2021.619013, PMID: 33767988 PMC7986718

[ref13] Corzo C. A. Condamine T. Lu L. Cotter M. J. Youn J. I. Cheng P.. (2010). HIF-1alpha regulates function and differentiation of myeloid-derived suppressor cells in the tumor microenvironment. J. Exp. Med. 207, 2439–2453. doi: 10.1084/jem.20100587, PMID: 20876310 PMC2964584

[ref14] Cui Y. Fang W. Li C. Tang K. Zhang J. Lei Y.. (2019). Development and validation of a novel signature to predict overall survival in "driver gene-negative" lung adenocarcinoma (LUAD): results of a multicenter study. Clin. Cancer Res. 25, 1546–1556. doi: 10.1158/1078-0432.CCR-18-2545, PMID: 30389658

[ref15] Dohlman A. B. Arguijo Mendoza D. Ding S. Gao M. Dressman H. Iliev I. D.. (2021). The cancer microbiome atlas: a pan-cancer comparative analysis to distinguish tissue-resident microbiota from contaminants. Cell Host Microbe 29, 281–298.e5. doi: 10.1016/j.chom.2020.12.001, PMID: 33382980 PMC7878430

[ref16] Dominguez-Bello M. G. Godoy-Vitorino F. Knight R. Blaser M. J. (2019). Role of the microbiome in human development. Gut 68, 1108–1114. doi: 10.1136/gutjnl-2018-317503, PMID: 30670574 PMC6580755

[ref17] Du X. Yang B. An Q. Assaraf Y. G. Cao X. Xia J. (2021). Acquired resistance to third-generation EGFR-TKIs and emerging next-generation EGFR inhibitors. Innovation (Camb) 2:100103. doi: 10.1016/j.xinn.2021.100103, PMID: 34557754 PMC8454558

[ref18] Du X. Zhang J. Wang J. Lin X. Ding F. (2018). Role of miRNA in lung Cancer-potential biomarkers and therapies. Curr. Pharm. Des. 23, 5997–6010. doi: 10.2174/1381612823666170714150118, PMID: 28714414

[ref19] Erb-Downward J. R. Thompson D. L. Han M. K. Freeman C. M. McCloskey L. Schmidt L. A.. (2011). Analysis of the lung microbiome in the "healthy" smoker and in COPD. PLoS One 6:e16384. doi: 10.1371/journal.pone.0016384, PMID: 21364979 PMC3043049

[ref20] Gahoi N. Syed P. Choudhary S. Epari S. Moiyadi A. Varma S. G.. (2020). A protein microarray-based investigation of cerebrospinal fluid reveals distinct autoantibody signature in low and high-grade gliomas. Front. Oncol. 10:543947. doi: 10.3389/fonc.2020.543947, PMID: 33415070 PMC7784397

[ref21] Ganapathy-Kanniappan S. Geschwind J. F. (2013). Tumor glycolysis as a target for cancer therapy: progress and prospects. Mol. Cancer 12:152. doi: 10.1186/1476-4598-12-152, PMID: 24298908 PMC4223729

[ref22] Garrett W. S. (2015). Cancer and the microbiota. Science 348, 80–86. doi: 10.1126/science.aaa4972, PMID: 25838377 PMC5535753

[ref23] Greathouse K. L. White J. R. Vargas A. J. Bliskovsky V. V. Beck J. A. von Muhlinen N.. (2018). Interaction between the microbiome and TP53 in human lung cancer. Genome Biol. 19:123. doi: 10.1186/s13059-018-1501-6, PMID: 30143034 PMC6109311

[ref24] Grunnet M. Sorensen J. B. (2012). Carcinoembryonic antigen (CEA) as tumor marker in lung cancer. Lung Cancer 76, 138–143. doi: 10.1016/j.lungcan.2011.11.012 22153832

[ref25] Guo Z. F. Kong F. L. (2021). Akt regulates RSK2 to alter phosphorylation level of H2A.X in breast cancer. Oncol. Lett. 21:187. doi: 10.3892/ol.2021.12448, PMID: 33574926 PMC7816342

[ref26] Hou H. Chen D. Zhang K. Zhang W. Liu T. Wang S.. (2022). Gut microbiota-derived short-chain fatty acids and colorectal cancer: ready for clinical translation? Cancer Lett. 526, 225–235. doi: 10.1016/j.canlet.2021.11.027, PMID: 34843863

[ref27] Hu X. Han T. Bian Y. Tong H. Wen X. Li Y.. (2020). Knockdown of SLCO4C1 inhibits cell proliferation and metastasis in endometrial cancer through inactivating the PI3K/Akt signaling pathway. Oncol. Rep. 43, 919–929. doi: 10.3892/or.2020.7478, PMID: 32020231 PMC7041124

[ref28] Huo Y. Zhou Y. Zheng J. Jin G. Tao L. Yao H.. (2022). GJB3 promotes pancreatic cancer liver metastasis by enhancing the polarization and survival of neutrophil. Front. Immunol. 13:983116. doi: 10.3389/fimmu.2022.983116, PMID: 36341459 PMC9627207

[ref29] Ianevski A. Giri A. K. Aittokallio T. (2022). Fully-automated and ultra-fast cell-type identification using specific marker combinations from single-cell transcriptomic data. Nat. Commun. 13:1246. doi: 10.1038/s41467-022-28803-w, PMID: 35273156 PMC8913782

[ref30] Jiang P. Gu S. Pan D. Fu J. Sahu A. Hu X.. (2018). Signatures of T cell dysfunction and exclusion predict cancer immunotherapy response. Nat. Med. 24, 1550–1558. doi: 10.1038/s41591-018-0136-1, PMID: 30127393 PMC6487502

[ref31] Lannering B. Marky I. Mellander L. Albertsson-Wikland K. (1988). Growth hormone secretion and response to growth hormone therapy after treatment for brain tumour. Acta Paediatr. Scand. Suppl. 77, 146–151. doi: 10.1111/j.1651-2227.1988.tb10815.x 3143223

[ref32] Le J. Fu Y. Han Q. Wei X. Ji H. Chen Y.. (2021). Restoration of mRNA expression of solute carrier proteins in liver of diet-induced obese mice by metformin. Front. Endocrinol. (Lausanne) 12:720784. doi: 10.3389/fendo.2021.720784, PMID: 34659115 PMC8515182

[ref33] Lee D. G. Lee J. H. Choi B. K. Kim M. J. Kim S. M. Kim K. S.. (2011). H(+)-myo-inositol transporter SLC2A13 as a potential marker for cancer stem cells in an oral squamous cell carcinoma. Curr. Cancer Drug Targets 11, 966–975. doi: 10.2174/156800911797264752 21861841

[ref34] Lindholm E. M. Leivonen S. K. Undlien E. Nebdal D. Git A. Caldas C.. (2019). miR-342-5p as a potential regulator of HER2 breast Cancer cell growth. Microrna 8, 155–165. doi: 10.2174/2211536608666181206124922, PMID: 30520388

[ref35] Liu X. S. Yang J. W. Zeng J. Chen X. Q. Gao Y. Kui X. Y.. (2022). SLC2A1 is a diagnostic biomarker involved in immune infiltration of colorectal Cancer and associated with m6A modification and ceRNA. Front. Cell Dev. Biol. 10:853596. doi: 10.3389/fcell.2022.853596, PMID: 35399515 PMC8987357

[ref36] Lopez-Siles M. Duncan S. H. Garcia-Gil L. J. Martinez-Medina M. (2017). *Faecalibacterium prausnitzii*: from microbiology to diagnostics and prognostics. ISME J. 11, 841–852. doi: 10.1038/ismej.2016.176, PMID: 28045459 PMC5364359

[ref37] Luo Y. Deng X. Que J. Li Z. Xie W. Dai G.. (2022). Cell trajectory-related genes of lung adenocarcinoma predict tumor immune microenvironment and prognosis of patients. Front. Oncol. 12:911401. doi: 10.3389/fonc.2022.911401, PMID: 35924143 PMC9339705

[ref38] Ma J. Sun L. Liu Y. Ren H. Shen Y. Bi F.. (2020). Alter between gut bacteria and blood metabolites and the anti-tumor effects of *Faecalibacterium prausnitzii* in breast cancer. BMC Microbiol. 20:82. doi: 10.1186/s12866-020-01739-1, PMID: 32272885 PMC7144064

[ref39] Maeta K. Hattori S. Ikutomo J. Edamatsu H. Bilasy S. E. Miyakawa T.. (2018). Comprehensive behavioral analysis of mice deficient in Rapgef2 and Rapgef6, a subfamily of guanine nucleotide exchange factors for rap small GTPases possessing the Ras/rap-associating domain. Mol. Brain 11:27. doi: 10.1186/s13041-018-0370-y, PMID: 29747665 PMC5946393

[ref40] Malta T. M. Sokolov A. Gentles A. J. Burzykowski T. Poisson L. Weinstein J. N.. (2018). Machine learning identifies Stemness features associated with oncogenic dedifferentiation. Cells 173, 338–354.e15. doi: 10.1016/j.cell.2018.03.034, PMID: 29625051 PMC5902191

[ref41] Marei H. E. Althani A. Afifi N. Hasan A. Caceci T. Felsani A.. (2022). Exome sequencing of glioblastoma-derived cancer stem cells reveals rare clinically relevant frameshift deletion in MLLT1 gene. Cancer Cell Int. 22:9. doi: 10.1186/s12935-021-02419-4, PMID: 34996478 PMC8740446

[ref42] Mariathasan S. Turley S. J. Nickles D. Castiglioni A. Yuen K. Wang Y.. (2018). TGFbeta attenuates tumour response to PD-L1 blockade by contributing to exclusion of T cells. Nature 554, 544–548. doi: 10.1038/nature25501, PMID: 29443960 PMC6028240

[ref43] Mima K. Nakagawa S. Sawayama H. Ishimoto T. Imai K. Iwatsuki M.. (2017). The microbiome and hepatobiliary-pancreatic cancers. Cancer Lett. 402, 9–15. doi: 10.1016/j.canlet.2017.05.001, PMID: 28527946

[ref44] Mirhadi S. Tam S. Li Q. Moghal N. Pham N. A. Tong J.. (2022). Integrative analysis of non-small cell lung cancer patient-derived xenografts identifies distinct proteotypes associated with patient outcomes. Nat. Commun. 13:1811. doi: 10.1038/s41467-022-29444-9, PMID: 35383171 PMC8983714

[ref45] Montealegre S. Lebigot E. Debruge H. Romero N. Heron B. Gaignard P.. (2022). FDX2 and ISCU gene variations Lead to rhabdomyolysis with distinct severity and Iron regulation. Neurol. Genet. 8:e648. doi: 10.1212/NXG.0000000000000648, PMID: 35079622 PMC8771665

[ref46] Najafi S. Abedini F. Azimzadeh Jamalkandi S. Shariati P. Ahmadi A. Gholami Fesharaki M. (2021). The composition of lung microbiome in lung cancer: a systematic review and meta-analysis. BMC Microbiol. 21:315. doi: 10.1186/s12866-021-02375-z, PMID: 34763672 PMC8582175

[ref47] Nakamura Y. Ochi Y. Satoh T. Satoh A. K. (2020). Rab10, crag and Ehbp1 regulate the basolateral transport of Na(+)K(+)ATPase in Drosophila photoreceptors. J. Cell Sci. 133:jcs238790. doi: 10.1242/jcs.238790, PMID: 32041903

[ref48] Orloff M. Peterson C. He X. Ganapathi S. Heald B. Yang Y. R.. (2011). Germline mutations in MSR1, ASCC1, and CTHRC1 in patients with Barrett esophagus and esophageal adenocarcinoma. JAMA 306, 410–419. doi: 10.1001/jama.2011.1029, PMID: 21791690 PMC3574553

[ref49] Pilaniya V. Gera K. Kunal S. Shah A. (2016). Pulmonary tuberculosis masquerading as metastatic lung disease. Eur. Respir. Rev. 25, 97–98. doi: 10.1183/16000617.00002315, PMID: 26929427 PMC9487663

[ref50] Poore G. D. Kopylova E. Zhu Q. Carpenter C. Fraraccio S. Wandro S.. (2020). Microbiome analyses of blood and tissues suggest cancer diagnostic approach. Nature 579, 567–574. doi: 10.1038/s41586-020-2095-1, PMID: 32214244 PMC7500457

[ref51] Rai A. Bleimling N. Vetter I. R. Goody R. S. (2020). The mechanism of activation of the actin binding protein EHBP1 by Rab8 family members. Nat. Commun. 11:4187. doi: 10.1038/s41467-020-17792-3, PMID: 32826901 PMC7442826

[ref52] Reck M. Remon J. Hellmann M. D. (2022). First-line immunotherapy for non-small-cell lung Cancer. J. Clin. Oncol. 40, 586–597. doi: 10.1200/JCO.21.01497 34985920

[ref53] Schabath M. B. Welsh E. A. Fulp W. J. Chen L. Teer J. K. Thompson Z. J.. (2016). Differential association of STK11 and TP53 with KRAS mutation-associated gene expression, proliferation and immune surveillance in lung adenocarcinoma. Oncogene 35, 3209–3216. doi: 10.1038/onc.2015.375, PMID: 26477306 PMC4837098

[ref54] Schotten L. M. Darwiche K. Seweryn M. Yildiz V. Kneuertz P. J. Eberhardt W. E. E.. (2021). DNA methylation of PTGER4 in peripheral blood plasma helps to distinguish between lung cancer, benign pulmonary nodules and chronic obstructive pulmonary disease patients. Eur. J. Cancer 147, 142–150. doi: 10.1016/j.ejca.2021.01.032, PMID: 33662689

[ref55] Seijo L. M. Peled N. Ajona D. Boeri M. Field J. K. Sozzi G.. (2019). Biomarkers in lung Cancer screening: achievements, promises, and challenges. J. Thorac. Oncol. 14, 343–357. doi: 10.1016/j.jtho.2018.11.023, PMID: 30529598 PMC6494979

[ref56] Sivan A. Corrales L. Hubert N. Williams J. B. Aquino-Michaels K. Earley Z. M.. (2015). Commensal Bifidobacterium promotes antitumor immunity and facilitates anti-PD-L1 efficacy. Science 350, 1084–1089. doi: 10.1126/science.aac4255, PMID: 26541606 PMC4873287

[ref57] Tort F. Ferrer-Cortes X. Thio M. Navarro-Sastre A. Matalonga L. Quintana E.. (2014). Mutations in the lipoyltransferase LIPT1 gene cause a fatal disease associated with a specific lipoylation defect of the 2-ketoacid dehydrogenase complexes. Hum. Mol. Genet. 23, 1907–1915. doi: 10.1093/hmg/ddt585, PMID: 24256811

[ref58] Vaupel P. Multhoff G. (2021). Revisiting the Warburg effect: historical dogma versus current understanding. J. Physiol. 599, 1745–1757. doi: 10.1113/JP278810, PMID: 33347611

[ref59] Vogtmann E. Goedert J. J. (2016). Epidemiologic studies of the human microbiome and cancer. Br. J. Cancer 114, 237–242. doi: 10.1038/bjc.2015.465, PMID: 26730578 PMC4742587

[ref60] Wang R. Liu H. Dong M. Huang D. Yi J. (2022). Exosomal hsa_circ_0000519 modulates the NSCLC cell growth and metastasis via miR-1258/RHOV axis. Open Med. (Wars) 17, 826–840. doi: 10.1515/med-2022-0428, PMID: 35582196 PMC9055259

[ref61] Wang G. Yu Y. Wang Y. Z. Wang J. J. Guan R. Sun Y.. (2019). Role of SCFAs in gut microbiome and glycolysis for colorectal cancer therapy. J. Cell. Physiol. 234, 17023–17049. doi: 10.1002/jcp.28436, PMID: 30888065

[ref62] Wang L. Zhou L. Li M. Zhao J. Liu Y. Chen Y.. (2022). Genome-wide CRISPR/Cas9 knockout screening uncovers ZNF319 as a novel tumor suppressor critical for breast cancer metastasis. Biochem. Biophys. Res. Commun. 589, 107–115. doi: 10.1016/j.bbrc.2021.12.023, PMID: 34902746

[ref63] Weersma R. K. Zhernakova A. Fu J. (2020). Interaction between drugs and the gut microbiome. Gut 69, 1510–1519. doi: 10.1136/gutjnl-2019-320204, PMID: 32409589 PMC7398478

[ref64] Weiss G. Schlegel A. Kottwitz D. Konig T. Tetzner R. (2017). Validation of the SHOX2/PTGER4 DNA methylation marker panel for plasma-based discrimination between patients with malignant and nonmalignant lung disease. J. Thorac. Oncol. 12, 77–84. doi: 10.1016/j.jtho.2016.08.123, PMID: 27544059 PMC5226366

[ref65] Werner S. Stamm H. Pandjaitan M. Kemming D. Brors B. Pantel K.. (2015). Iroquois homeobox 2 suppresses cellular motility and chemokine expression in breast cancer cells. BMC Cancer 15:896. doi: 10.1186/s12885-015-1907-4, PMID: 26560478 PMC4642646

[ref66] Xia L. Mei J. Kang R. Deng S. Chen Y. Yang Y.. (2022). Perioperative ctDNA-based molecular residual disease detection for non-small cell lung Cancer: a prospective multicenter cohort study (LUNGCA-1). Clin. Cancer Res. 28, 3308–3317. doi: 10.1158/1078-0432.CCR-21-3044, PMID: 34844976

[ref67] Xu Y. Chai B. Wang X. Wu Z. Gu Z. Liu X.. (2022). miRNA-199a-5p/SLC2A1 axis regulates glucose metabolism in non-small cell lung cancer. J. Cancer 13, 2352–2361. doi: 10.7150/jca.67990, PMID: 35517408 PMC9066207

[ref68] Xu L. Deng C. Pang B. Zhang X. Liu W. Liao G.. (2018). TIP: a web server for resolving tumor Immunophenotype profiling. Cancer Res. 78, 6575–6580. doi: 10.1158/0008-5472.CAN-18-0689, PMID: 30154154

[ref69] Xu W. L. Liu S. Li N. Ye L. F. Zha M. Li C. Y.. (2021). Quercetin antagonizes glucose fluctuation induced renal injury by inhibiting aerobic glycolysis via HIF-1alpha/miR-210/ISCU/FeS pathway. Front Med (Lausanne) 8:656086. doi: 10.3389/fmed.2021.656086, PMID: 33748166 PMC7969708

[ref70] Xu Y. R. Wang A. L. Li Y. Q. (2022). Hypoxia-inducible factor 1-alpha is a driving mechanism linking chronic obstructive pulmonary disease to lung cancer. Front. Oncol. 12:984525. doi: 10.3389/fonc.2022.984525, PMID: 36338690 PMC9634253

[ref71] Xu X. Zhao Y. Ying Y. Zhu H. Luo J. Mou T.. (2023). m7G-related genes-NCBP2 and EIF4E3 determine immune contexture in head and neck squamous cell carcinoma by regulating CCL4/CCL5 expression. Mol. Carcinog. 62, 1091–1106. doi: 10.1002/mc.23548, PMID: 37067401

[ref72] Yan C. Niu Y. Ma L. Tian L. Ma J. (2022). System analysis based on the cuproptosis-related genes identifies LIPT1 as a novel therapy target for liver hepatocellular carcinoma. J. Transl. Med. 20:452. doi: 10.1186/s12967-022-03630-1, PMID: 36195876 PMC9531858

[ref73] Yan X. Yang M. Liu J. Gao R. Hu J. Li J.. (2015). Discovery and validation of potential bacterial biomarkers for lung cancer. Am. J. Cancer Res. 5, 3111–3122. PMID: 26693063 PMC4656734

[ref74] Yoshihara K. Shahmoradgoli M. Martinez E. Vegesna R. Kim H. Torres-Garcia W.. (2013). Inferring tumour purity and stromal and immune cell admixture from expression data. Nat. Commun. 4:2612. doi: 10.1038/ncomms3612, PMID: 24113773 PMC3826632

[ref75] Yu G. Gail M. H. Consonni D. Carugno M. Humphrys M. Pesatori A. C.. (2016). Characterizing human lung tissue microbiota and its relationship to epidemiological and clinical features. Genome Biol. 17:163. doi: 10.1186/s13059-016-1021-1, PMID: 27468850 PMC4964003

[ref76] Yu Y. Liu Q. Li W. Qu Y. Zhang Y. Liu T. (2020). Identification of a novel EHBP1-MET fusion in an intrahepatic cholangiocarcinoma responding to Crizotinib. Oncologist 25, 1005–1008. doi: 10.1634/theoncologist.2020-0535, PMID: 32897609 PMC7938406

[ref77] Zhang Y. Foreman O. Wigle D. A. Kosari F. Vasmatzis G. Salisbury J. L.. (2012). USP44 regulates centrosome positioning to prevent aneuploidy and suppress tumorigenesis. J. Clin. Invest. 122, 4362–4374. doi: 10.1172/JCI63084, PMID: 23187126 PMC3533537

[ref78] Zhang D. Jiang Q. Ge X. Shi Y. Ye T. Mi Y.. (2021). RHOV promotes lung adenocarcinoma cell growth and metastasis through JNK/c-Jun pathway. Int. J. Biol. Sci. 17, 2622–2632. doi: 10.7150/ijbs.59939, PMID: 34326698 PMC8315012

[ref79] Zhou Y. Guo Y. Wang Y. (2022). Identification and validation of a seven-gene prognostic marker in colon cancer based on single-cell transcriptome analysis. IET Syst. Biol. 16, 72–83. doi: 10.1049/syb2.12041, PMID: 35352485 PMC8965382

[ref80] Zhou J. B. Zhang T. Wang B. F. Gao H. Z. Xu X. (2013). Identification of a novel gene fusion RNF213-SLC26A11 in chronic myeloid leukemia by RNA-Seq. Mol. Med. Rep. 7, 591–597. doi: 10.3892/mmr.2012.1183, PMID: 23151810

