# Supplementary material for: Intratumor microbiome derived glycolysis-lactate signatures depicts immune heterogeneity in lung adenocarcinoma by integration of microbiomic, transcriptomic, proteomic and single-cell data
Source: Front Microbiol. 2023 Aug 17;14:1202454. doi: 10.3389/fmicb.2023.1202454 (PMC10469687; doi:10.3389/fmicb.2023.1202454)
Supplement: Supplementary file 1 [file Data_Sheet_1.docx]

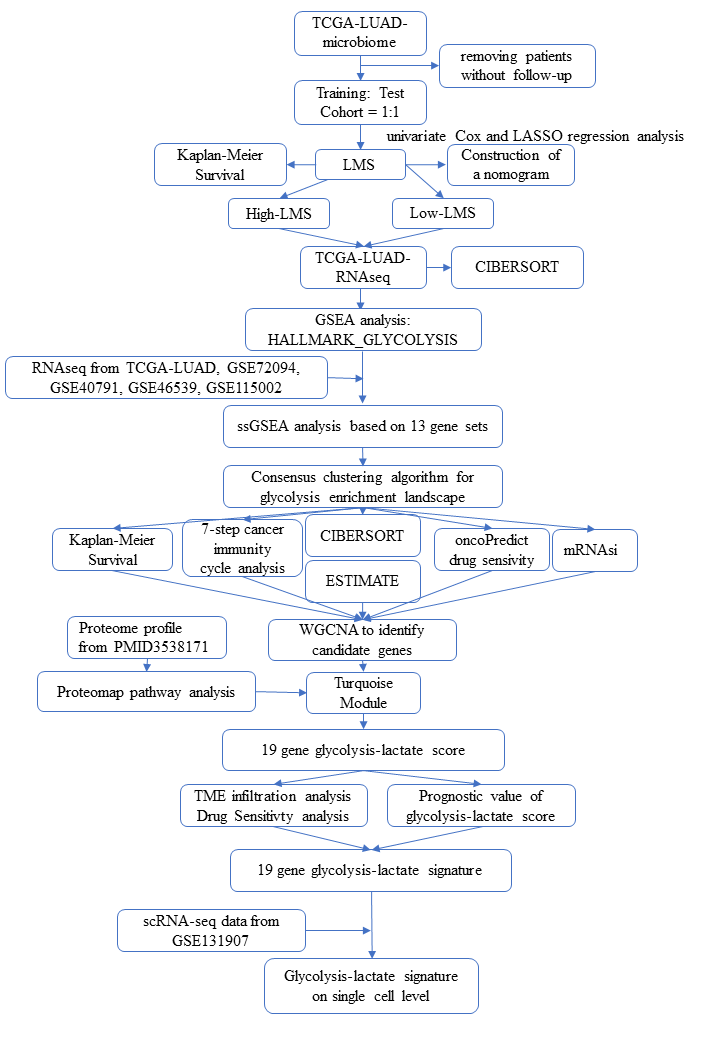


**Supplement Figure 1. Flow chart of this study.**


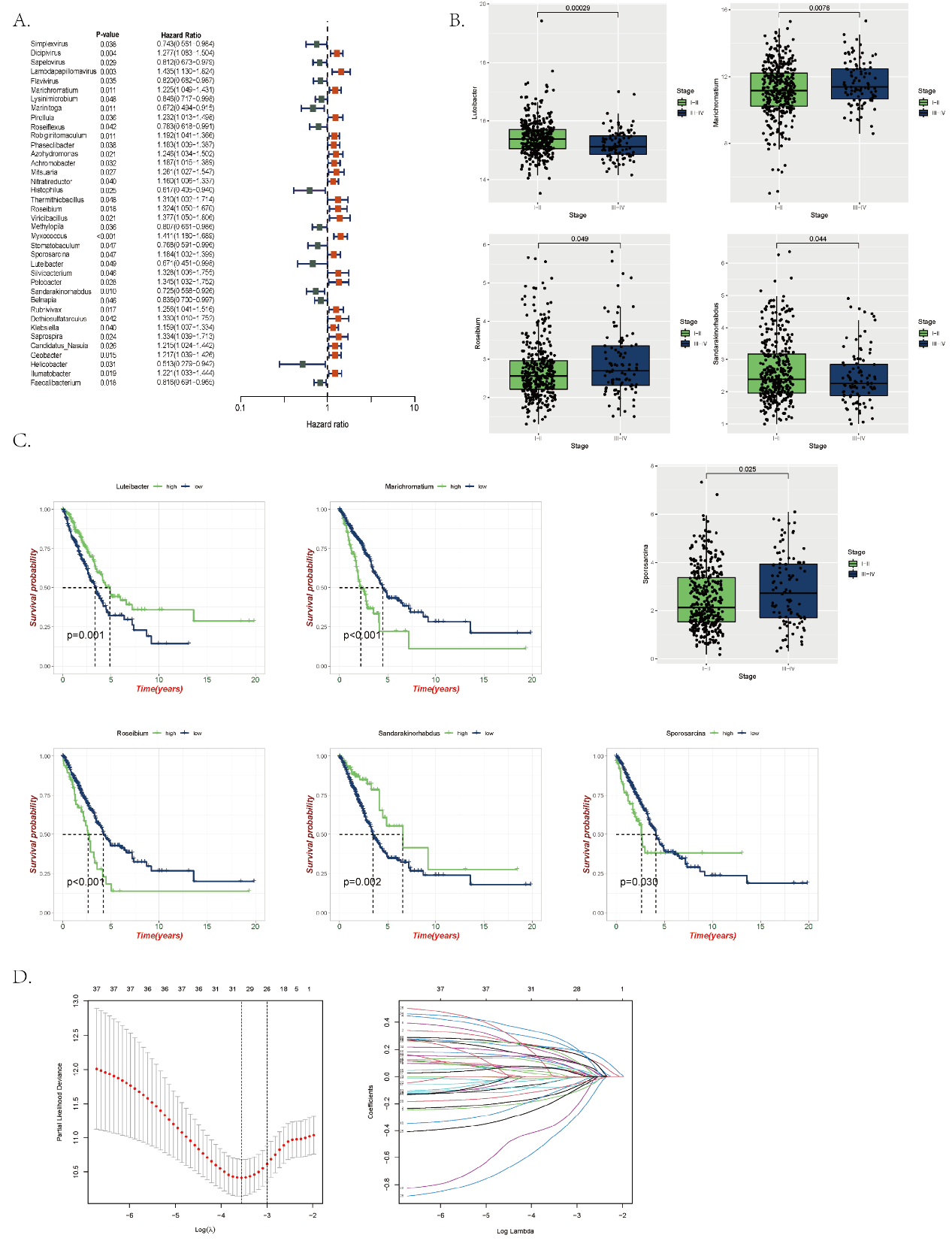


**Supplement Figure 2. Association between microbial abundance and overall survival (OS).** (A) Forest plot illustrates the hazard ratio for each prognostic microbe using multivariate Cox regression. (B) Box plots show the association between abundance of microbes and tumor stages. (C) Kaplan-Meier OS curves for LUAD patients are presented based on the abundance of representative microbes. (D) Optimal tunning parameter and LASSO efficient profile of candidate microbes.

**
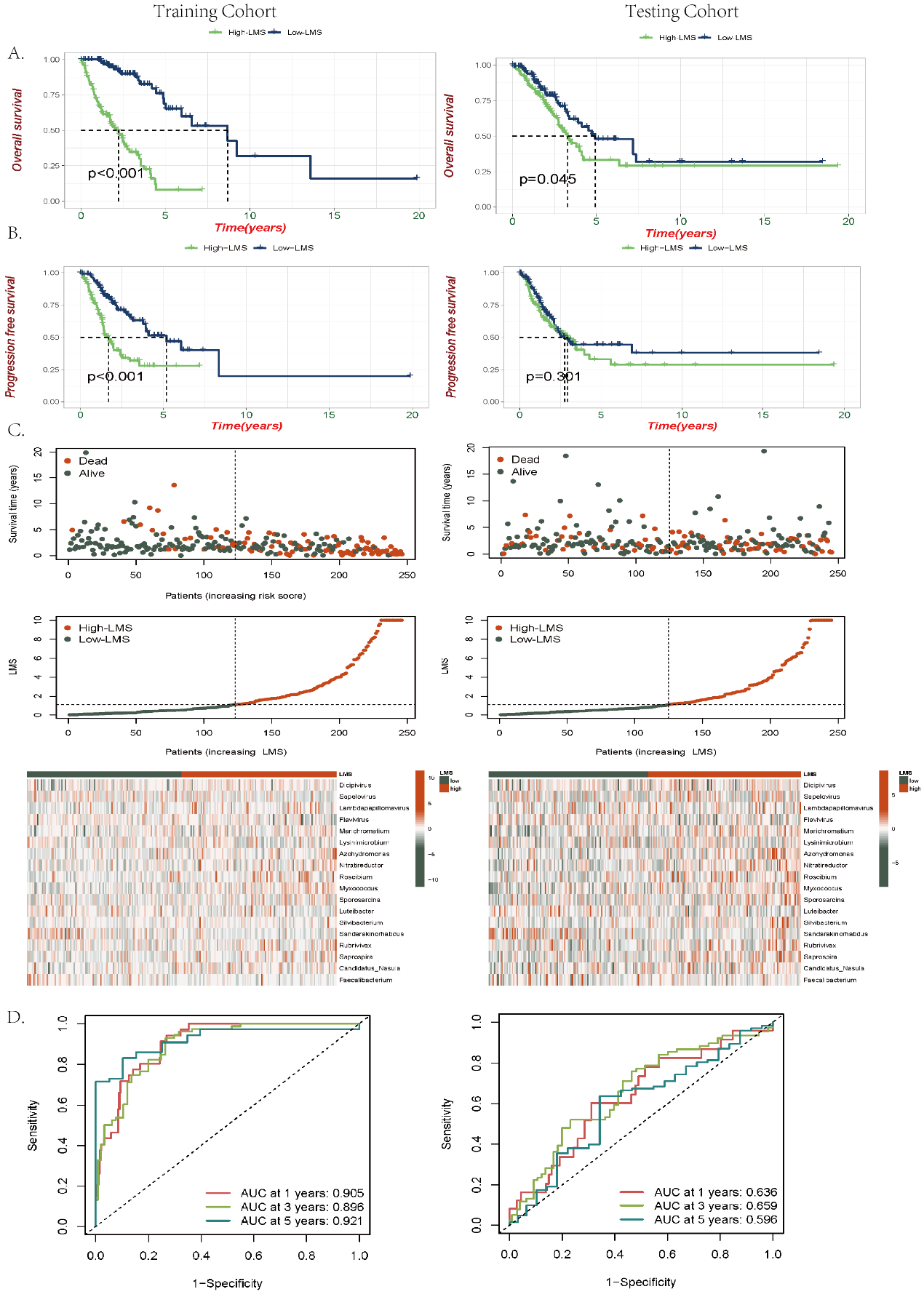

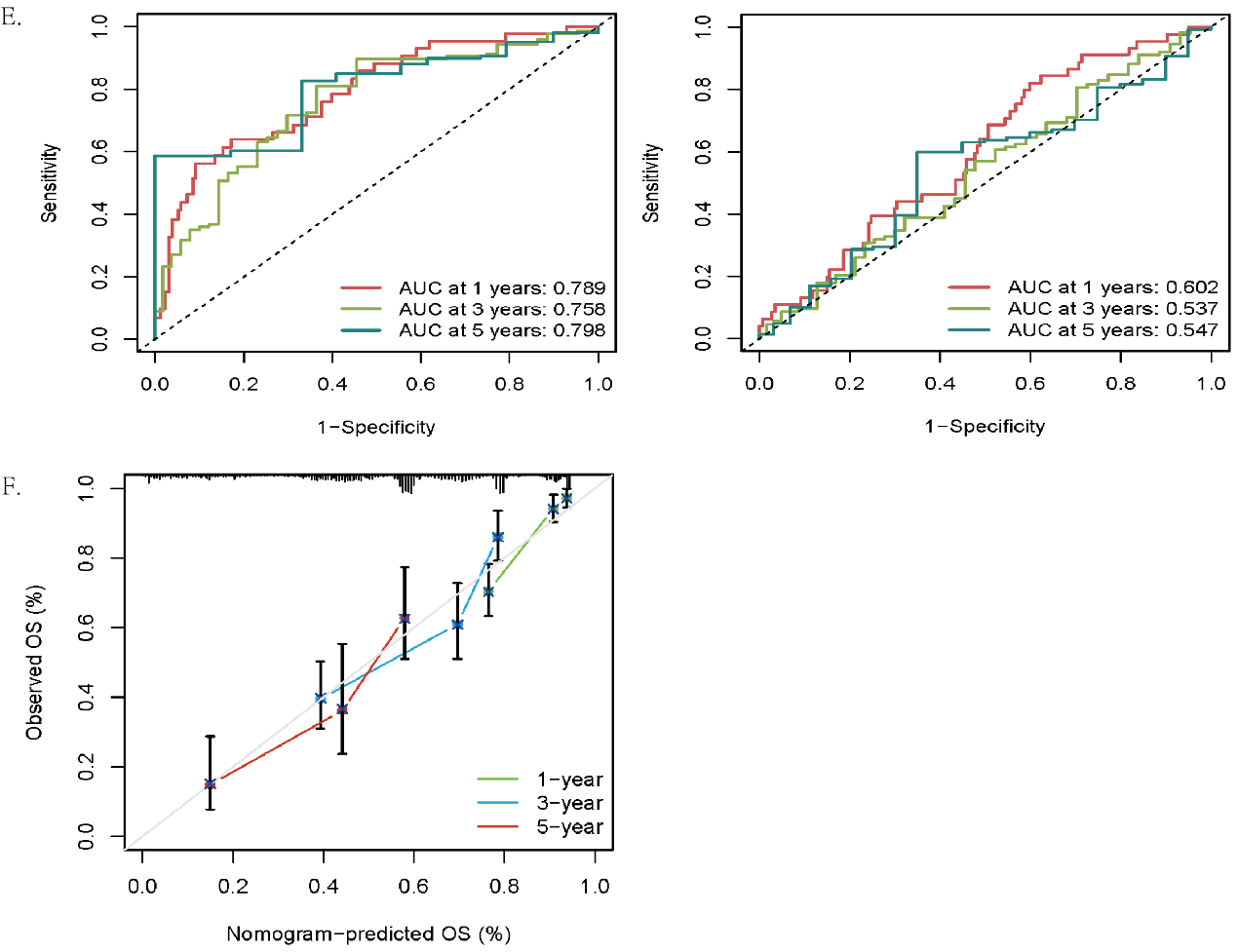
**

**Supplement Figure 3. Lung-resident microbial score construction.** (A) Kaplan-Meier analysis of overall survival (OS) in training cohort and testing cohort. (B) Kaplan-Meier analysis of progression free survival (PFS) in training cohort and testing cohort. (C) The distribution of survival status, risk scores and abundance of 18 prognostic microbes in training cohort and testing cohort. (D) LMS predicting OS using time-independent ROC analysis in training cohort and testing cohort. (E) LMS predicting PFS using time-independent ROC analysis in training cohort and testing cohort. (F) Calibration curves for the LMS nomogram for 1, 3 and 5 years.

**
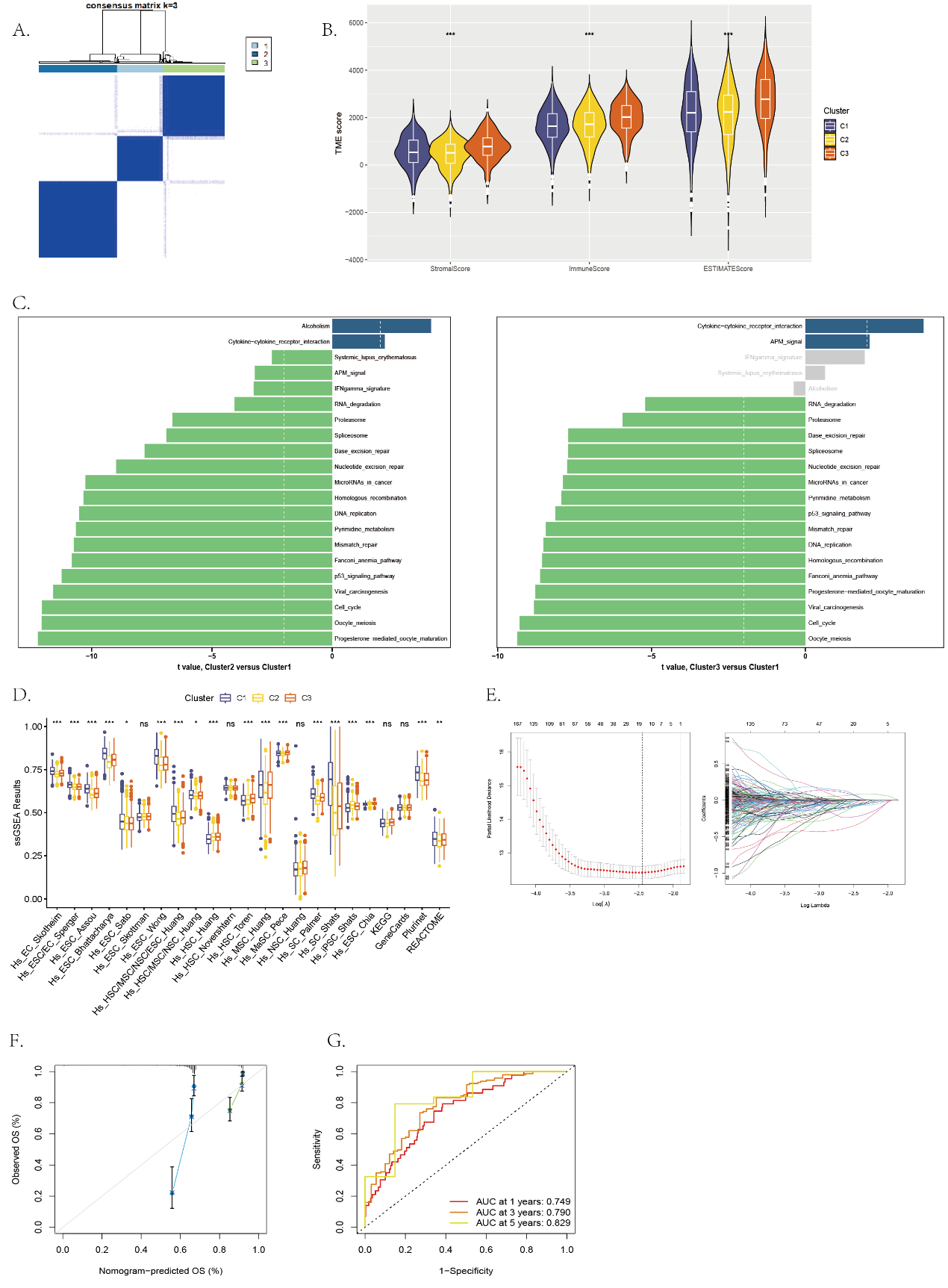
**

**Supplement Figure 4. Immune characteristics among glycolysis and lactate metabolic patterns in LUAD. (A) Three distinct clusters were produced through the application of unsupervised clustering procedures utilizing 13 glycolysis and lactate metabolic gene sets. (B) Comparisons of the three clusters in stromal score, immune score and estimate score. (C) Differences in immunotherapy efficacy related pathways were shown between Cluster 2 versus Cluster 1 (Left) and Cluster 3 versus Cluster 1 (Right). (D) Comparisons of the three clusters in tumor stemness gene sets. (E) Optimal tunning parameter and LASSO efficient profile of candidate genes to build glycolysis-lactate score. (F) Calibration curves for the glycolysis-lactate score nomogram for 1, 3 and 5 years. (G) LMS predicting OS using time-independent ROC analysis.**

**
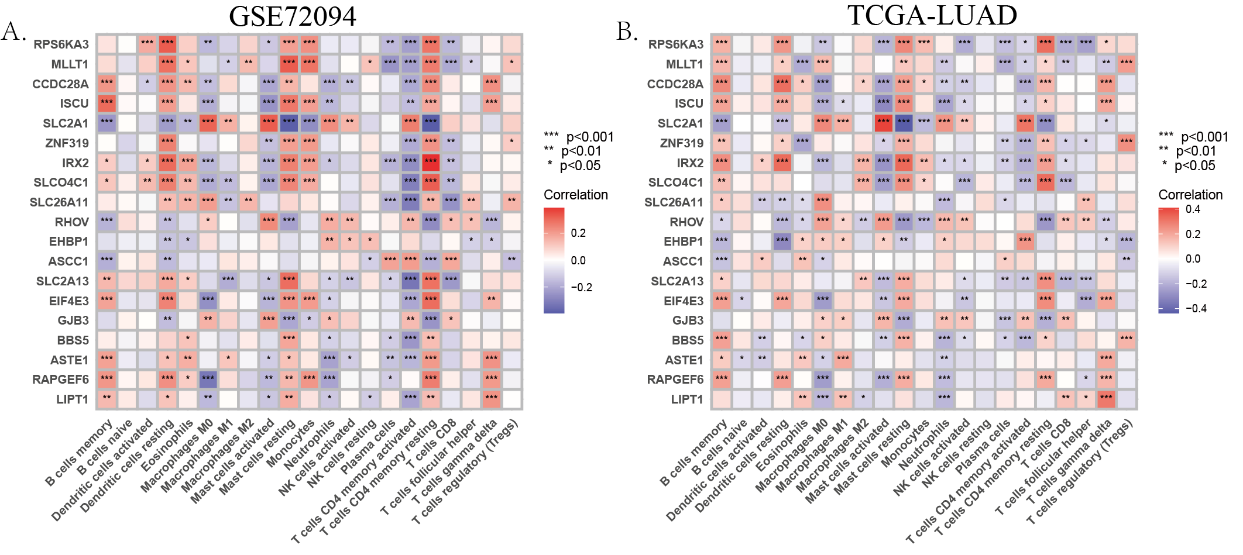
**

**Supplement Figure 5. Correlation between 14 novel genes and immune cell infiltration in GSE72094 (A) and TCGA-LUAD cohort (B) respectively.**

**
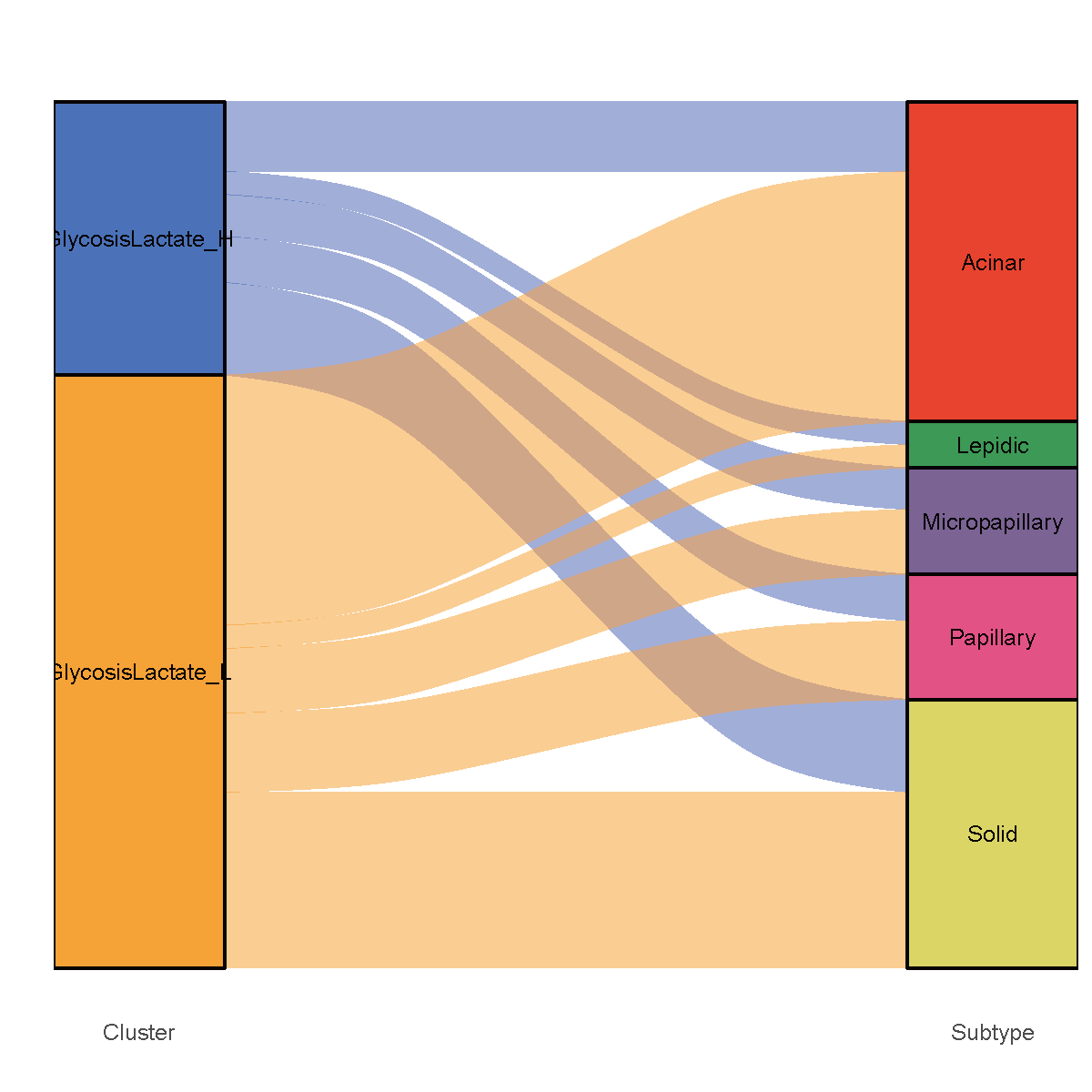
**

**Supplement Figure 6. A Sankey diagram was illustrated to visualize the relationship between glycolysis-lactate signature and histological subtypes in TCGA dataset.**
